# Chronic *Chlamydia* infection in human organoids increases stemness and promotes age-dependent CpG methylation

**DOI:** 10.1038/s41467-019-09144-7

**Published:** 2019-03-18

**Authors:** Mirjana Kessler, Karen Hoffmann, Kristin Fritsche, Volker Brinkmann, Hans-Joachim Mollenkopf, Oliver Thieck, Ana Rita Teixeira da Costa, Elena I. Braicu, Jalid Sehouli, Mandy Mangler, Hilmar Berger, Thomas F. Meyer

**Affiliations:** 1 Department of Molecular Biology, Max Planck Institute for Infection Biology, Charitéplatz 1, 10117 Berlin, Germany; 2 Department of Gynecology, Charité University Medicine, Campus Virchow, Augustenburger Platz 1, 13353 Berlin, Germany; 3 Department of Gynecology and Obstetrics, Auguste-Viktoria-Klinikum, Rubensstr. 125, 12157 Berlin, Germany

## Abstract

Chronic infections of the fallopian tubes with *Chlamydia trachomatis* (*Ctr*) cause scarring and can lead to infertility. Here we use human fallopian tube organoids and genital *Ctr* serovars D, K and E for long-term in vitro analysis. The epithelial monolayer responds with active expulsion of the bacteria into the lumen and with compensatory cellular proliferation—demonstrating a role of epithelial homeostasis in the defense against this pathogen. In addition, *Ctr* infection activates LIF signaling, which we find to be an essential regulator of stemness in the organoids. Infected organoids exhibit a less differentiated phenotype with higher stemness potential, as confirmed by increased organoid forming efficiency. Moreover, *Ctr* increases hypermethylation of DNA, which is an indicator of accelerated molecular aging. Thus, the chronic organoid infection model suggests that *Ctr* has a long-term impact on the epithelium. These heritable changes might be a contributing factor in the development of tubal pathologies, including the initiation of high grade serous ovarian cancer.

## Abstract (web summary)

Chronic infections of the fallopian tubes with *Chlamydia trachomatis* can cause scarring and infertility. Here, the authors show that the pathogen alters stem cell differentiation and DNA methylation in human fallopian tube organoids, suggesting a potential link to cellular ageing and malignant transformation.

## Introduction

Understanding the mechanisms of fallopian tube (FT) homeostasis and pathology constitutes an important medical challenge, particularly in light of women’s fertility and beyond. Notably, the FT is the likely tissue of origin of high-grade serous ovarian cancer (HGSOC), the deadliest gynecological malignancy^1^. Yet, progress in this area has been painstakingly slow, due to the absence of suitable experimental models as well as the lack of diagnostic tools. Among the most common causes of tubal pathology is the Gram negative pathogen *Ctr*, affecting 1.6 million people in 2016 in the USA alone^2^. It frequently leads to chronic infections due to a lack of symptoms and can result in tubal scarring and occlusion, the major cause of infertility and ectopic pregnancy^3,4^.

As an obligate intracellular pathogen, *Ctr* has been shown in vitro to subvert host cell metabolism, block apoptosis, and impact genome integrity by causing DNA damage and triggering degradation of p53^5–7^. Nevertheless, key steps in the development of *Ctr*-induced tubal inflammation (salpingitis) remain obscure, including the natural progression of infections. In particular, there is a gap in our knowledge concerning the long-term consequences of *Ctr* infections on epithelial homeostasis. Several early studies reported structural damage to the FT^8,9^. Previously, we also showed that several paracrine pathways are activated in response to acute *Ctr* infection ex vivo^10^, suggesting the existence of broader host defense mechanisms that include both infected and neighboring non-infected cells. Still, it has so far been impossible to analyze the molecular sequels of these initial events during the establishment of chronic *Ctr* infections in the human model. It is also unclear if protracted microbial colonization of the tube adds to the risk of cellular transformation and ovarian cancer development, since the epidemiological data remain inconsistent^11,12^.

Establishment of long-term organoid cultures from human primary FT epithelial stem cells^13^ has created an opportunity for a qualitatively new approach to study pathogen–host interactions during *Ctr* infection. Longevity of the organoids, genetic stability, preserved differentiation mechanisms, and high structural similarity of the organoid monolayer to the epithelium in vivo are all essential components of the model, making it an ideal system to investigate the molecular mechanisms of chronic *Ctr* infection. *Ctr* has 15 different serovars, which can be divided into three categories: A–C, which cause ocular disease, D–K, which cause urogenital infections, and L1–L3, which cause invasive lymphoma granuloma venerum (LGV).

Here, we report the establishment of a chronic *Ctr* infection model of FT organoids with genital serovars D, K, and E, which are the major drivers of tubal pathology in vivo. We identify sustained pathogen-driven changes in cellular differentiation of the epithelium that occur over the course of 9 months of infection, showing that *Ctr* not only alters the phenotype of host cells but also leaves a lasting mark in the epigenome.

## Results

### Human FT organoids as a model of chronic *Ctr* infection

**Fig. 1:**
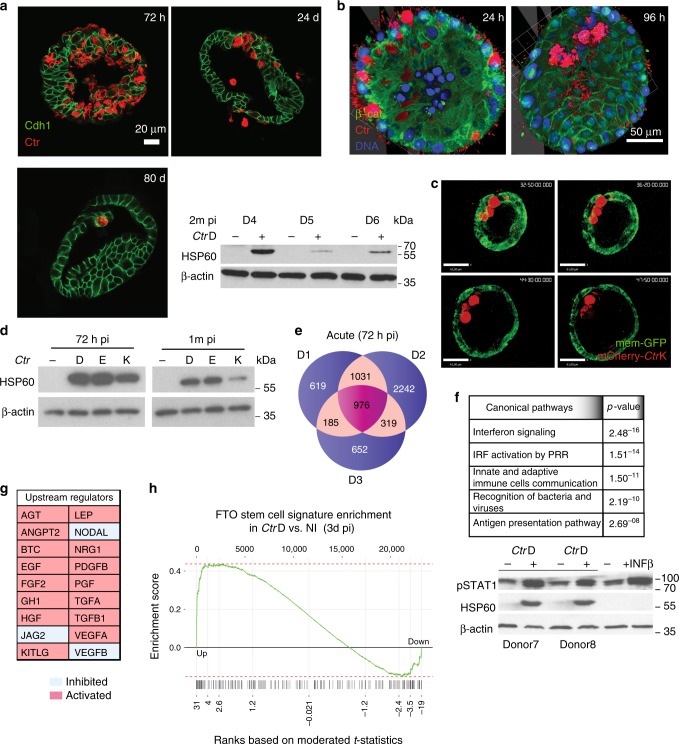
Chronic *Ctr* infection of hFT organoids is characterized by early activation of inflammatory and paracrine signaling responses. **a** Representative confocal images of organoids infected with *CtrD* at 3, 24, and 80 d p.i., showing continuing presence of bacterial inclusions (red) within epithelial host cells (green). Scale bar: 20 µm. The WB of protein lysates from chronic infection at 2 months p.i. from three independent donors shows the presence of actively replicating bacteria (HSP60 protein). **b** Whole-mount 3D composite images of acute *Ctr* infection at 34 and 96 h p.i. showing a change in the localization of the bacteria (*Ctr*, red) from the inclusions within the primarily infected host cells (left panel) to the lumen of the organoid (right panel) (β-catenin, green). Scale bar: 50 µm. **c** Still images as a time course from Supplementary Movie 1 demonstrate stepwise extrusion of bacterial inclusions to the lumen. Scale bar: 60 µm. **d** Different genital *Ctr* serovars D, K, and E establish chronic infection in the organoids and maintain growth at 1 month p.i., as shown in the WB of the protein lysates harvested at acute and chronic phase. **e** Venn diagram showing extensive overlap between three different donors in the genes differentially expressed in response to *Ctr* infection, as detected by microarray analysis at 72 h p.i., with 976 genes regulated in all samples. **f** Ingenuity pathway analysis, suggesting strong activation of pro-inflammatory signaling, in particular interferon-β, which was independently confirmed by WB and detection of phospho STAT1 in infected samples with interferon-β treated organoids as positive control. **g** Table of activated paracrine pathways in the acute *Ctr* infection based on ingenuity upstream regulator analysis IPA. **h** GSEA analysis of fallopian tube (FT) organoid stem cell signature genes^13^ compared to differential gene expression in infected vs. non-infected organoids at 3 d p.i. shows significant enrichment (ES = 0.44, *p* < 0.001, permutation test using 5000 permutations) among upregulated genes

We used organoid cultures from human FTs, as described previously^13^, for infection with *Ctr* serovar D (*Ctr*D) and investigated its effect on epithelial homeostasis. In stark contrast to conventional infection models based on transformed cell lines, which allow *Ctr* propagation for only a single life cycle due to lysis of infected cells, the organoids accommodated the bacteria for extended periods of time and continued to expand at a normal rate, despite an ongoing productive infection. Immunofluorescence analyses suggested that ~30% of cells were initially infected. The life cycle duration of ~72 h did not detectably differ from that typically observed for *Ctr* in cell lines, as indicated by confocal microscopy at 3 d post-infection (p.i.; Fig. 1a). Confocal analysis at later time points revealed that large *Chlamydia* inclusions were still present in organoids at 1 and even 3 months p.i, although their numbers greatly decreased during that time (Fig. 1a). Actively replicating bacteria were detected in protein lysates at 3 days, 1 month, and 2 months, but at >4 months p.i. no signal was present, as judged by western blot analysis *of Ctr* HSP60 protein in relation to host cell actin levels in total cell lysates of infected organoids (Supplementary Figure 1a). The presence of chronic infection at 2 months p.i. was further validated in three independent donor cultures (Fig. 1a, WB panel). The infectious potential was determined by infectivity assay at 72 h and 1 month p.i. Successful infection of HeLa cells with retrieved elementary bodies (EBs) (Supplementary Figure 1b) at both time points proved that *Ctr* is able to complete multiple life cycles within the organoids and stay infectious over time. The titer of retrieved bacteria did gradually decrease, however, indicating the presence of defense mechanisms that contain and eventually eliminate the infection. We observed no large variation in retrieved bacteria between different donors, quantified by number of EBs/cell (Supplementary Figure 1b). Notably, organoid cultures infected with 10 µl of 4 × 10^8^ (CFU ml^−1^) *Ctr* EBs always survived, and we have successfully propagated chronically infected organoids for >3 months in 20 of 20 cases, indicating the robustness of this model. Overall, the long-term expandability of infected organoid cultures was maintained for >1 year, indicating that *Ctr* infection did not impair the stemness potential of the organoids. We wanted to analyze the dynamics of acute *Ctr* infection in organoids in more detail, including its progression beyond the first 72–96 h. Whole-mount labeling of the infected organoids, which enables a comprehensive 3D view of the epithelial structure, revealed large *Ctr* inclusions within the monolayer at 24 h p.i. In contrast, at 96 h p.i. the bacteria were predominantly located in the organoid lumen, suggesting the existence of a clearance mechanism (Fig. 1b).

To visualize these events in real time, live-cell imaging was performed from 24 to 72 h p.i. on an organoid line with pCT-Mem-GFP-labeled membranes infected with an mCherry-labeled *Ctr* strain. This revealed a fast and dynamic process of expulsion of intact *Ctr* inclusions and/or infected cells from the epithelial layer into the lumen, where they subsequently burst to release infective EBs (Fig. 1c, Supplemental Movie 1). Interestingly, this phenotype was the dominant route of resolution of *Ctr*-host interaction in the organoids, resulting in an almost complete preservation of epithelial integrity. This is evidence of an intact tissue defense mechanism that limits damage to the host epithelium and enables survival of the organoid. In stark contrast to this, the well-described phenotype of *Ctr* infection in monolayers results in widespread death of host cells, such that only induction of *Ctr* persistency by antibiotics can extend the life span of infected cultures beyond 3–4 days. The concurrent robust expulsion of the inclusions and/or infected cells we observed and the maintenance of an intact polarized monolayer with no detectable structural damage also suggest that pathways that respond to changes in cell density, tissue organization and mechano-transduction are involved in maintaining epithelial integrity during the infection. To test whether the observed infection pattern is similar for the other serovars clinically relevant for urogenital infections, we also infected organoids with *Ctr* K and E strains and confirmed the presence of replicative bacteria at 1 month p.i. by western blotting for hsp60 protein (Fig. 1d). This finding was further validated by immunofluorescence imaging (Supplementary Figure 1c), which revealed no differences in the dynamics of inclusion growth and inclusion shedding between the different strains. Therefore, we conclude that chronic organoid infection is likely to reflect the interaction of *Ctr* with the FT epithelium in vivo, regardless of the strain.

### Gene expression profile of acute infection in the organoids

To investigate the global host–cell response during the acute phase of the infection, microarray analysis was performed on organoid cultures from three different donors infected with *Ctr*D for 72 h, while technical replicates were kept in long-term culture. Comparative analysis of differentially expressed genes revealed a largely similar response between different donors (Fig. 1e) to *Ctr* challenge, characterized by strong upregulation of large networks of genes involved in inflammation, pathogen recognition receptors, and communication between innate and adaptive immunity (Fig. 1f). Robust IFN-β signaling was the most prominent hallmark of acute infection, with the majority of known network components upregulated (Supplementary Figure 2a). Western blotting of whole-cell lysates from organoids confirmed that the main mediator of IFN-β signaling, the transcription factor STAT1, was strongly phosphorylated upon *Ctr* infection (Fig. 1f, lower panel) at levels comparable to that induced by stimulation with exogenous IFN-β. Interestingly, despite the absence of immune cells in our model, our microarray data revealed a strong upregulation of inducible nitric oxide synthase (NO2) (Supplementary Figure 2b) after *Ctr* infection, which was previously reported to depend on induction by activated T cells^14,15^, suggesting that the epithelium responds to *Ctr* infection with a cell-autonomous, broad activation of inflammatory pathways. Importantly, in addition to the activation of regulatory networks involved in the innate immune response, we also observed differential regulation of important mediators of paracrine signaling, epithelial differentiation, and homeostasis. Analysis of upstream regulators using the Ingenuity Pathway Analysis IPA software platform identified activated (red) and inhibited (white) networks triggered by potent growth factors, such as EGF, PDGHFB, and TGF-β (Fig. 1g), with a wide range of functions that include control of cell growth, proliferation, and cell survival. As key regulators of epithelial homeostasis, these pathways have proved to be essential for control of epithelial integrity, response to injury, and wound healing in numerous other tissues, thus their activation in response to *Ctr* illustrates the extent of pathogen–host interaction in the organoids. In addition, we observed a strong upregulation of the leukemia inhibitory factor (LIF) signaling pathway, including LIF itself, as well as its downstream targets, e.g., OCT4, SOCS1, TNF-α, and IGF3BP^16^ (Supplementary Figure 3), which suggests changes in both differentiation and inflammation. In confirmation, gene set enrichment analysis (GSEA) (Fig. 1h) comparing the acute infection response to a published set of NOTCH-regulated stemness genes detected in FT organoids^10^ revealed a significant enrichment in the group of upregulated genes, suggesting that at 3 days p.i. organoids exhibit an increase in stemness. Taken together, acute *Ctr* infection of FT organoids triggers a sustained innate inflammatory response coupled with activation of homeostatic mechanisms to repair the injury. These findings are in agreement with the observed phenotypic changes in organoids at early stages of the infection process. Newly infected cultures are characterized by the presence of cellular debris derived from apoptotic cells, as confirmed by the presence of activated caspase-3 during the first 48 h (Supplementary Figure 4a), likely initiated by an early stress response. The absence of caspase-3 activation at 72 h, a time point when intense shedding of inclusions and/or cells occurs, confirms that infected cells do not die by apoptosis. Indeed, confocal images of infected organoids revealed that active caspase-3 signal does not co-localize with inclusion-bearing cells (Supplementary Figure 4b), thus cell death is likely triggered by a paracrine mechanism in the neighbouring uninfected cells.

### Infected organoids regain homeostasis by increased proliferation

**Fig. 2:**
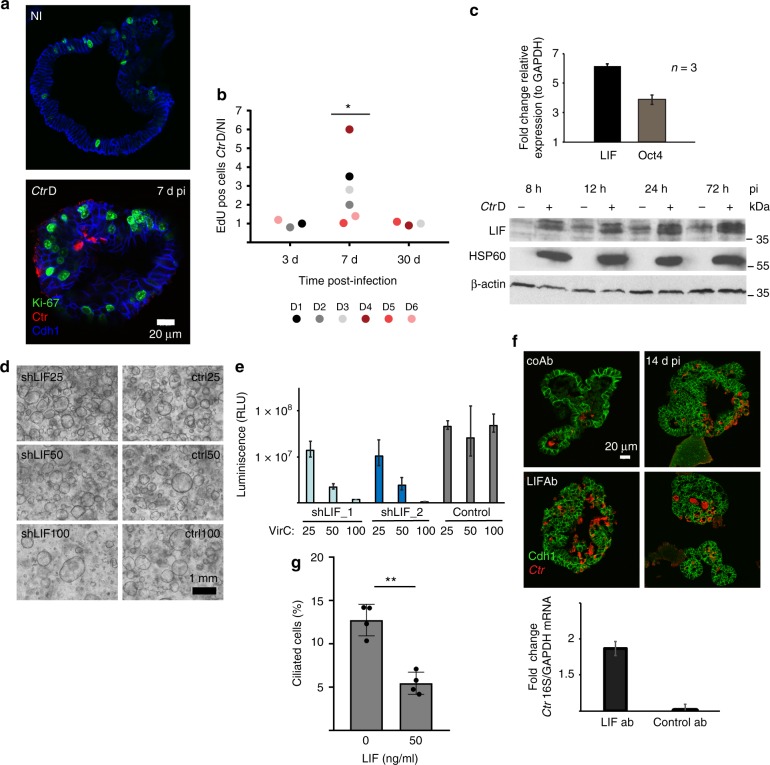
LIF controls organoid homeostasis and limits replication of *Ctr* in the epithelium. **a** Confocal images showing that in infected organoids actively proliferating Ki67+ cells (green) are more frequent in cells neighboring infected cells (Ctr: red, Cdh1: blue) at 7d p.i. Scale bar: 20 µm. **b** Quantification of the time course experiments from 6 *Ctr* infections (Donors D1-D6), by number of EdU-positive cells in the infected sample, as detected by FACS normalized to the control. Infected organoids exhibit a transient peak in proliferation at one week p.i., while long-term growth rate, measured at 30 days p.i. remains constant. Significance was determined by paired Student’s *t*-test of EdU measurement data sets in non-infected (NI) and infected (*Ctr*D) conditions. **c** Upregulation of LIF and its target gene Oct4 in acute *Ctr* infection was validated by qPCR in three independent donors at 72 h p.i. Data are presented as mean ± sd. Induction of LIF starts from the early phase of *Ctr* infection, as increased protein levels are already detected at 8 h p.i. by western blot analysis of the first 3 days. **d** Phase contrast images showing that lentiviral transduction of shRNA against LIF (at 25, 50, or 100 of viral units) greatly reduces the number of generated organoids in a dose-dependent manner. Ctrl: control vector pLKO.5. **e** The effect on organoid formation upon LIF knockdown by using different concentrations of virus particles (VirC) was quantified by ATP-based viability assay, which detects luminescence signal. Error bars represent technical variation between triplicate wells ± SD. **f** Inhibition of LIF activation by neutralizing antibody leads to increased bacterial load (*Ctr* red, Cdh1 green) detected in confocal images at 14 d p.i.; scale bar: 20 µm. The difference was quantified by qPCR at the same time point as the change in abundance of bacterial 16S relative to GAPDH level of the host cells. Data are representative of two independent experiments and presented as mean ± sd of technical replicates. CoAb: control antibody against *H. pylori* Cag A protein. **g** Active LIF, at 50 ng/ml promotes a secretory phenotype in the organoids as evidenced by reduction in the number of ciliated cells. Data are representative of two independent experiments. Quantification is performed as number of ciliated cells/nuclei in five independent fields of view (dot plot) (>1000 nuclei/sample) ± sd represents variation between individual images. **p < 0.01 (*p* = 0.0057) was calculated by paired Student’s *t*-test

In contrast to the acute infection, chronically infected organoids phenotypically resemble controls (Supplementary Figure 4c). Long-term infected cultures also maintain a constant expansion rate over many passages and are passaged at the same time points and ratios as their non-infected sister organoids over the course of many months. We therefore hypothesized that newly infected organoids undergo increased proliferation in order to compensate for the initial cell loss. Analysis of confocal images at 7 d p.i. revealed increased numbers of Ki67+ cells in infected cultures. Positive nuclei were predominantly present in non-infected cells in close proximity to infected cells (Fig. 2a). FACS-based analysis over the course of one month to determine the proportion of EdU-labeled cells in infected vs uninfected organoids from six different donors revealed a significant increase in the proliferation rate, with a peak at 7 d p.i. (Fig. 2b, Supplementary Figure 5a). By 30 d, the proliferation rate in infected organoids had returned to control levels, in congruence with the observed lack of differences in the long-term expansion rate. This suggests that in order to maintain epithelial integrity, non-infected cells transiently increase their expansion rate to replace infected cells that have been extruded from the epithelium. Nevertheless, the observed robust activation of paracrine networks controlling not only cell growth and proliferation but also differentiation and cell fate in general, suggests that *Ctr* infection has pervasive long-term consequences on the organoid epithelium. We therefore went on to analyze pathways with dual functions that could potentially connect inflammatory responses with those involved in epithelial homeostasis.

### LIF signaling regulates organoid formation and differentiation

LIF belongs to the IL-6 family of cytokines and is one of the major regulators of pluripotency during early embryogenesis as well as a crucial determinant of uterine receptivity during implantation in humans. In addition, it has potent anti-inflammatory effects, regulating IL-1β, IL-6, IL-7, IL-2Rα, and IFN-γ in a cutaneous inflammation model^17^ and limiting the degree of lung injury from *E. coli* infection^18^. Our microarray data revealed that acute *Ctr* infection consistently triggers LIF upregulation in all three donors. In agreement with this, increased expression of the LIF target gene OCT4 in infected vs non-infected cells was confirmed by qPCR (Fig. 2c, upper panel). Western blot analysis further validated these findings, by revealing increased amounts of LIF protein from as early as 8 h until at least 72 h p.i. (Fig. 2c, lower panel). Considering the functional importance of LIF in preserving stemness in other models, we were interested in whether its induction during acute *Ctr* infection could contribute to infection-driven changes in homeostasis.

Interestingly, western blot analysis showed that phospho-STAT3, the main downstream effector of LIF, was constitutively active in FT organoids (after 1 d post-seeding), while the passaging procedure induced a transient dephosphorylation that lasted for 8 h, indicating changes in the STAT3 LIF/STAT3 signaling axis during organoid development, which is reinitiated after every passage (Supplementary Figure 5b). This suggests that the LIF/STAT3 axis may also be involved in the regulation of epithelial homeostasis and differentiation in the FT. Indeed, depletion of LIF transcript via shRNA greatly decreased organoid-forming capacity in epithelial isolates with only few organoids forming (Fig. 2d). Depending on the virus titer, the number of organoids generated dropped to <2% of that observed after transduction with control vector, as quantified by the Cell Titer-Glo® assay, which measures the number of live cells via the ATP content (Fig. 2e). The organoids that did grow still expressed basal levels of LIF protein that were the same between cultures transduced with different amounts of virus particles, suggesting that LIF is essential for organoid growth in vitro (Supplementary Figure 5c). Therefore, we conclude that LIF has a central role during organoid formation, and thus ultimately controls epithelial renewal in the FT. Interestingly, blocking LIF pathway activation with soluble neutralizing antibody at the time of infection increased the chlamydial load in the organoids (Fig. 2f), as visualized by immunofluorescence analysis at 14 d p.i. and confirmed in a separate experiment by qPCR of *Ctr* 16s (Fig. 2f). While activation of LIF signaling thus limits replication of the pathogen, we were interested in whether it also affects the organoid phenotype. Adding recombinant LIF to non-infected cultures resulted in a reduced percentage of ciliated cells, quantified as the number of cilia per nuclei (Fig. 2g), suggesting that amplification of the LIF signal alone is sufficient to shift the balance of cell types within the epithelium towards a less differentiated, secretory phenotype.

### Chronic infection leads to increased stemness in organoids

**Fig. 3:**
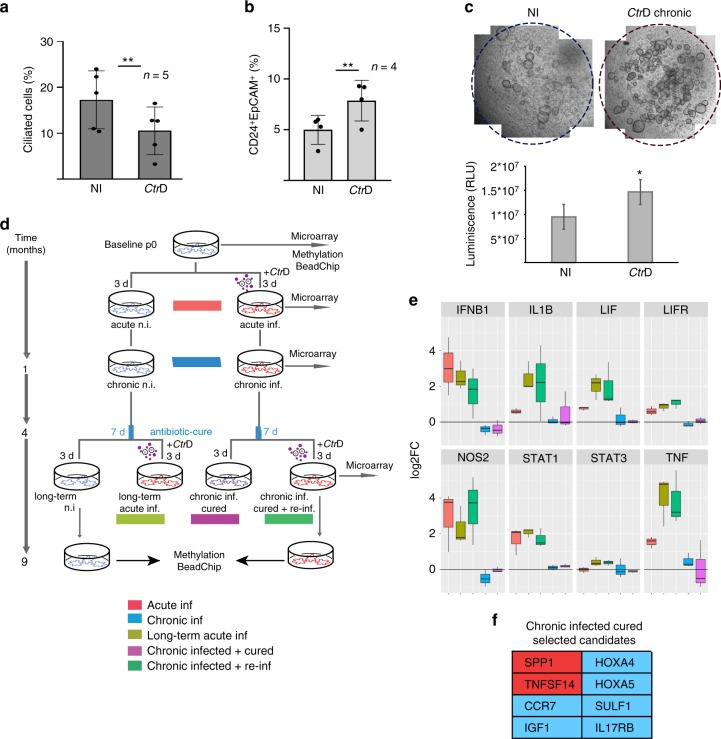
Chronic *Ctr* infection leads to fewer ciliated cells, increased stemness markers and downregulation of immunomodulators. **a** Chronic infection (>3 months) significantly reduces the number of ciliated cells in the organoids. Percentage of ciliated cells was calculated as the number of ciliated cells per nuclei per field of view. Data represent mean ± SD from five independent experiments; dot plot reflects data points from independent infections **p = 0.0059, paired Student’s *t*-test. **b** Chronically infected organoids show increased numbers of CD24+/EpCam+ cells, as determined by FACS profiling. Data represent mean ± SD of four independent chronic infections. Data points from independent experiments are represented on dot plot. **p = 0.0071 paired Student’s *t*-test. **c** Chronic *Ctr* infection causes increase in organoid forming efficiency as visible on phase contrast images of comparative *Ctr*+/− wells of organoids grown from respective single cells suspensions. The effect was quantified by Cell Titer-Glo® assay. *p < 0.05, paired Student’s *t*-test, calculated from measurements of technical replicates. Data are representative of three independent experiments. **d** Experimental outline of acute/chronic infections, indicating time points at which samples for microarray and methylation analysis were collected. For each infected sample, the non-infected control sample at the same time point served as control. **e** Differential expression of selected genes across acute (3 d p.i.), chronic (1 m p.i.), and cured (4 m p.i.) infection experiments compared to non-infected control (as determined by microarray analysis) shows a conserved inflammatory response to *Ctr*, which subsides at later stages of infection and after clearance of the pathogen. Samples are colour-coded as in **d**, each was compared to the non-infected control culture at the same time point, as shown in **d**. Box plots show median, quartiles, maximum, and minimum of the log2 fold-changes of three donors. **f** Selected genes from the table of jointly regulated candidates (Supplementary Data 1) that remained differentially regulated after curing of chronic infection (red: upregulated, blue: downregulated). Genes include regulators of extracellular signaling (SPP1, SULF1), developmental genes (HOX4A and HOX5A), and immunomodulators (CCR7 and IL17 RB)

The robust changes in signaling and in the regulation of cell fate and differentiation factors during the acute phase of the infection suggested that *Ctr* infection potentially has long-lasting consequences on the epithelium. Quantification of the cell types found in chronically infected cultures by analysis of confocal images of serial sections, revealed that the percentage of ciliated cells significantly decreased by 3 months p.i., as determined by the number of cilia/nuclei compared to non-infected control cultures in chronic infections of organoids from five different donors (Fig. 3a). To test whether this reduction is linked to an increase in the proportion of less differentiated cells, we used FACS to analyze the number of cells expressing stem cell surface markers. Indeed, chronic infection led to an increase in the number of CD24^+^/EpCam^+^ cells (Fig. 3b, Supplementary Figure 6a), in four different donors, in agreement with the general shift towards a less differentiated state. Stem cells of both healthy and diseased upper genital tract tissue have previously been defined as CD24^+^/EpCam^+^^19^. In addition, in 2 out of 3 donors, infected organoids contained an increased proportion of cells positive for CD133, a surface marker that is associated with an increase in stemness potential^20^ (Supplementary Figure 6b). Thus, both cell-type composition of the organoids, as well as surface-marker distribution, strongly suggest changes in the homeostasis of chronically infected cultures compared to controls. To test whether there is also a difference in stemness potential, we dissociated organoids to single cells and quantified their organoid forming efficiency. Indeed, cells from chronically infected organoids had a significantly higher organoid forming capacity, as quantified by Cell Titer-Glo 3D viability assay (Fig. 3c). Thus, we conclude that chronic *Ctr* infection leads to increased stemness of the organoid epithelium. Expansion of the secretory cell phenotype in the FT has been postulated as a cornerstone of transformation during the development of HGSOC^1^. In line with our previous observations in FT organoids^13^, lineage tracing in mice has recently confirmed that secretory cells are in fact the precursors of ciliated cells^21^. Therefore, the *Ctr*-induced expansion of the secretory phenotype due to decreased terminal differentiation may be a potential risk factor for the initiation of sequels that could lead to transformation.

To obtain comprehensive insight into the signaling events that characterize long-term *Ctr* infection in the organoid model, we generated exploratory data sets at multiple time points during chronic infection of organoids from independent donors. As the strongest risk factor for *Ctr*-driven pathology is known to be the number of episodes of recurrent disease or reinfections, we added groups to examine whether such infections could facilitate long-lasting or even heritable changes—which would likely be mediated via epigenetic CpG methylation. To this end, we carried out global gene expression analysis at 72 h and 1 month p.i., and also at 4 months p.i. At this point, cultures were cured and re-infected (chronic infected + reinfected) and compared to the acute infection of organoids from the non-infected arm kept in culture from the start of the experiment (long-term, acute infected) (Fig. 3d). To ensure any residual bacteria were cleared prior to reinfection, all cultures were treated with antibiotics, and the absence of viable bacteria confirmed by infectivity assay (Supplementary Figure 7a). Finally, re-infected organoids were maintained in culture for an additional 4 months and analyzed for changes in DNA methylation in comparison to the methylation profile of non-infected parallel control cultures as well as “baseline” organoid cultures from the same donors, from which genomic DNA had been isolated at passage 0.

The gene expression pattern of organoids infected for 4 months followed by 7 days of antibiotic treatment (chronic infected + cured) compared to control organoids of the same age (long-term non-infected) showed that a number of genes remained dysregulated beyond resolution of infection. We identified 91 significantly regulated genes between cured and never infected samples (log2 fold change <−0.7 or >0.7, *p* < 0.01 with an FDR < 61%; Supplementary Data 1. Statistics based on moderated *t*-test and adjusting *p*-values for multiple testing using Benjamini–Hochberg procedure). Interestingly, one of the genes that was consistently upregulated in cultures from all three donors was osteopontin (SPP1) (Fig. 3f). This secreted matricellular protein can mediate migration and cell survival and is involved in the pathogenesis of multiple chronic diseases involving cancer and chronic inflammation^22^. Also, chronically infected, cured organoids show sustained overexpression of TNFSF14 (LIGHT), a member of the TNF family of ligands. Among the downregulated genes was the homeobox gene HOXA4, a prognostic factor in ovarian cancer that suppresses growth and cell motility^23^, as well as HOXA5^24^, which has been shown to prevent cellular transformation and positively regulates p53 expression. Downregulated genes with major roles in the modulation of the adaptive immunity included ILR17RB receptor, CCR7 or KLRF1.

Interestingly, despite these differences, we did not observe relevant changes in the cellular responses to acute *Ctr* infection between organoids that were previously chronically infected and cured, and control cultures (short or long term) that had not been infected previously. In particular, major immune response effectors like IFN-β1, TNF, IL-1β, LIF, STAT1, and NOS1 were strongly upregulated in all acute infection conditions, while chronically infected (1 month) and cured samples did not show such changes (Fig. 3e), suggesting that inflammatory responses subsided. This likely reflects the progressive reduction of bacterial numbers in the organoids over time, as confirmed by qPCR of bacterial 16S rRNA (Supplementary Figure 7b).

Thus, we concluded that while previous chronic *Ctr* infection does not change the immediate response of the fallopian epithelium to a fresh acute challenge, it does cause prolonged changes in the cellular composition of the organoid monolayer and the distribution of stemness markers. Moreover, there is evidence that chronic *Ctr* infection alters the interaction between the epithelium and the microenvironment, due to changes in expression levels of active extracellular proteins (SPP1, SULF1, IGF1) and immunomodulators (CCR7, IL17 RB). This finding could be of great importance for understanding the development and clinical sequels of *Ctr*-driven pathology, especially in the context of recurrent episodes of asymptomatic salpingitis.

### CpG hypermethylation is increased upon chronic infection

To understand how chronic *Ctr* infection leads to long-lasting changes in gene expression even after the infection is cured, we examined whether it affects the epigenome of host cells. To identify DNA methylation pattern changes, genomic DNA from organoids cultured for a total of 9 months, which were cured and re-infected at 4 months (Fig. 3d), was analyzed using the Illumina Infinium® Methylation EPIC BeadChip, covering >850,000 CpGs of the human genome. We first tried to identify changes across three independent donors between long-term infected and non-infected conditions. Applying a threshold of at least 20% differential methylation and a *p*-value of 0.05, we identified 603 CpGs at a minimal false discovery rate of 76%, of which 179 were located in promoter regions (Supplementary Data 2). For most genes, only a single CpG out of many represented on the array was affected, indicating that there was no strong deterministic effect of long-term infection on DNA methylation compared to control.

**Fig. 4:**
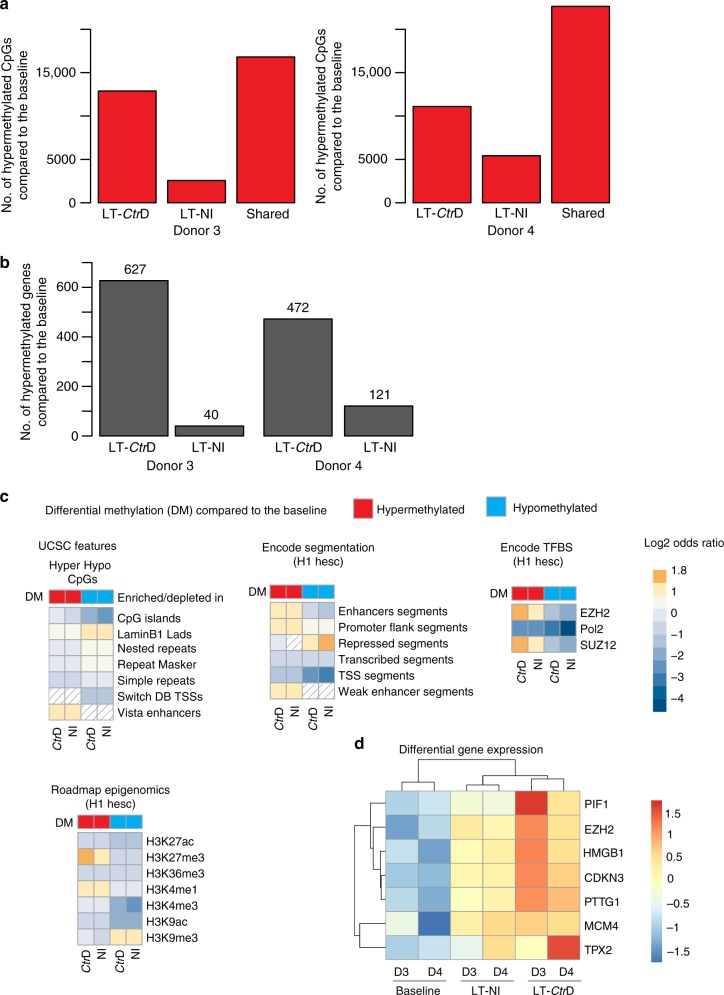
Impact of chronic *CtrD* infection on the methylome. **a** Barplots showing the numbers of CpGs that increase in DNA methylation (hypermethylation; delta beta > 0.2) specifically in long-term (LT; 9 months; compare experimental design in Fig. 3c) infected (*Ctr*D) or non-infected (NI) samples compared to baseline, as well as those which are hypermethylated in both arms of the experiment (shared) in donor 3 (left) and donor 4 (right). **b** Barplot displaying the number of genes where hypermethylation, specifically for infected or non-infected samples compared to the baseline, affects at least three CpGs/gene. **c** Heatmaps of enriched and depleted genomic features among differentially methylated CpGs. The color code indicates a significant enrichment (orange) or depletion (blue) based on the log2 odds ratio and a FDR < 5%. No significant enrichment or depletion (FDR > 5%) is displayed by dashed squares. DM differential methylation, H1 hesc H1 human embryonic stem cell line, TFBS transcription factor binding sites. **d** Heatmap representing the differential expression of selected genes comparing baseline, long-term (9 months) non-infected, and infected organoids. The standardized gene expression data (*z*-score) of two patients (D3 and D4) were obtained from single-color microarray analysis

As stochastic processes have been implicated in the formation of differentially methylated regions^25^, we hypothesized that infection might lead to stochastic changes in DNA methylation that may not be detectable in organoid cultures due to the limited number of stem cells that contribute to long-term maintenance. We thus analyzed samples from two different donors to determine differentially methylated CpGs in both infected and non-infected samples, as compared to the baseline DNA methylation profile of the parental organoid cultures. We used data from a control experiment on gastric primary cells to define the magnitude of relevant changes in the absence of replicates (Supplementary Figure 8a, b) and concluded that methylation changes of >20% in any direction most likely represent real biological changes. Using this threshold, ~10–12% of all CpGs on the array were found to be differentially methylated during the course of the experiment. Notably, while changes in DNA methylation were detected in both arms of the experiment, infected samples showed an increased number of hypermethylated CpGs in both donors (Fig. 4a) compared to non-infected samples (*χ*^2^ test, *p* < 0.001 for both replicates, Supplementary Figure 8c), leading to an increase in the number of hypermethylated genes (Fig. 4b). In order to determine whether there are any common functional denominators or distribution patterns among affected CpGs, data were subjected to Locus overlap analysis (LOLA)^26^. Identification of the genomic regions where changes in the DNA methylation occurred revealed that differentially methylated CpGs are not randomly distributed. Globally, they are enriched for laminin B1-associated domains (LAD) and depleted for transcriptionally active genomic regions, including transcription start sites (TSS), transcribed segments, and for CpG islands (Fig. 4c). Instead, hypermethylated CpGs in both conditions were enriched for enhancer and promoter flanking sites, as well as regions that, in human embryonic stem cells, are known to bind the polycomb repressive complex 2 (PRC2) members EZH2 and SUZ12—and to show histone modification H3K27me3^27^. Regulation of DNA methylation by PRC2 and hypomethylation of repetitive genomic regions have both been implicated in the process of molecular ageing across a number of sectional studies^28–30^. To analyze if the detected methylation changes correlate with the gene expression profiles of aged organoid cultures, we have performed microarray analysis of infected and non-infected long-term cultures at 9 months from both donors. At this time point, we could not conclusively confirm a significant correlation, which could be due to the small sample size or the inherent complexity of the regulatory mechanisms that mediate the effect of individual CpGs on gene transcription. Still, we detected distinct changes in the gene expression profile of long-term cultures over time, and upregulation of numerous genes involved in packaging of DNA, chromosome assembly and segregation (K4H20 demethylase network), regulation of replication (MCM4 and MCM10), control of cell cycle (BUB1B, CDKN3) and process of cellular ageing (EZH2, PIF1). All these markers provide a broader context regarding the set of molecular changes at the genomic level that characterize prolonged cultivation of adult stem cells. Interestingly, the heatmap of candidate genes (Fig. 4d) shows a tendency for higher expression levels in the infected cultures compared to non-infected aged controls, which also supports the conclusion that infection promotes the process of aging.

Taken together, our findings demonstrate sustained age-related changes in DNA methylation during a longitudinal study in a controlled experimental model. Thus, organoids appear to undergo an in vitro ageing process that is highly similar to that observed in vivo, further validating the authenticity of this model. Importantly, although there are no apparent qualitative differences in the pattern of CpG changes between experimental arms—indicating that the same underlying process is at work—chronic *Ctr* infection appears to accelerate this process in vitro. Interestingly, while the gene expression data of acute *Ctr* infection suggests an initial downregulation of the EZH2 transcription factor and corresponding target genes, long-term culture leads to sustained upregulation of EZH2. Overexpression of EZH2 is frequently detected in HGSOC tissue and is a potential therapeutic target^31^. Further studies are needed to determine whether there is a causative relationship between the transcriptional changes in EZH2 and the observed differences in CpG methylation. Altogether, our data provide experimental evidence that chronic *Ctr* infection causes long-term changes in homeostasis of the host epithelium, gene expression, and the epigenetic landscape, which are maintained beyond the end of the infection.

## Discussion

Previous investigations of the effects of *Ctr* infection on human host cells were performed in 2D models mostly using transformed cell lines. Such models correlate poorly with the structural complexity and cell–cell communication that are key features of the healthy epithelial lining in the genital tract. Importantly, due to the aggressive nature of the *Ctr* lytic cycle in monolayer culture, it is also impossible to follow the process of infection long-term without induction of persistence. However, it is chronic and asymptomatic *Ctr* infections that are the main drivers of pathology in the upper genital tract. Thus, there is a great medical need for developing new *Ctr* infection models in order to understand the molecular basis of disease development.

Analysis of the long-term consequences of both acute and chronic ascending infections in vivo is greatly impeded by the inaccessibility of the FTs to clinical diagnostic tools. Instead, past infections are not usually documented until years later, and based solely on the evidence of positive *Ctr* serology and structural damage to the tube. Our chronic *Ctr* infection model now provides unique insights into the nature and cellular mechanisms of *Ctr*-driven pathogenesis in a human primary FT epithelium that closely resembles the characteristics of the native tissue. We show that chronic infection can be established in organoids with all tested *Ctr* genital serovars (D, K, E), which underlines the validity of the model for studying the general mechanisms of *Ctr* infection and for investigating the mechanisms that lead to the associated pathology. Our data were generated by thorough long-term analysis of expression data from organoid cultures from 4 different donors, and analysis of methylation changes in organoids from 3 donors, infected with *Ctr* for 9 months. It thus offers a first insight into the events that characterize the long-term effects of chronic *Ctr* infection. In addition to data presented here, which originate from 10 donors, we have performed additional chronic infection experiments (>3 months), all of which followed a consistent pattern in terms of course of infection—confirming the robustness of the infection model and the conserved response to *Ctr* infections, regardless of genetic background.

Our results indicate a profound, yet homeostatic, effect of *Ctr* infection on the regulation of cell fate and differentiation. By using organoids, it was possible to demonstrate that the epithelium limits the physical damage resulting from infection by efficiently extruding inclusions and infected cells into the lumen. While rupture of the inclusion membrane appears to occur mostly after extrusion from the monolayer, we could not conclusively determine the fate of the infected cells. However, it is clear that *Ctr* bacteria are able to maintain a prolonged infection in the organoid epithelium, during which shedding into the lumen is followed by fresh cycles of infection and active bacterial replication. The diminishing *Ctr* titer over time is evidence of a successful defense mechanism of the host epithelium. However, we cannot rule out that it could also reflect limitations of the model, e.g., the inevitable loss of infectious EBs into the medium when organoids are broken up for passaging.

Although the acute infection leads to activation of multiple paracrine growth factors (TGF-β1, EGF, FGF etc.), we identify LIF as a key player in the maintenance of stemness in tubal organoids. This underscores the fundamental role of LIF signaling in the female genital tract, as evidenced by the fact that LIF mutant mice are sterile and have an underdeveloped uterus^32^. The *Ctr*-driven amplification of LIF signaling we observed suggests a mechanistic molecular basis for the link between salpingitis and the risk of ectopic pregnancy, which has previously been connected to overexpression of LIF in the FT^33^. Due to the dual function of the LIF signaling network in regulating stemness^34,35^ and mediating anti-inflammatory effects, it is possible that the peak in LIF activity is primarily triggered by innate immune mechanisms in order to limit bacterial replication, as evidenced by our pharmacological inhibition of LIFR. Stemness-related changes may be a by-product in the course of chronic infection.

The observed gradual decrease in the number of ciliated cells and increase in CD24+Epcam+ and CD133+ cells suggest a sustained shift in the regulation of epithelial renewal during infection. This effect establishes a link towards cell transformation, since expansion of non-ciliated, secretory cells generally precedes HGSOC development^1^. It is important to note that the increase in stemness, as measured by the increased organoid forming efficiency, does not imply complete functional competence of the ageing stem cells, as previously demonstrated in other models^36^. Equally important may be our observation that chronically infected organoids show changes in the expression of the potent immunomodulators IL17 RB, CCR7, and KLRF1, and that this effect persists after the clearance of the bacteria. In context with the detected upregulation of SPP1 and reduction of HOXA4 and HOXA5, this may modify the way the epithelium interacts with the surrounding tissue, including adaptive immune cells. However, determining the precise implications of these findings will require more extended experimental settings, involving immune cells as well as larger numbers of donors to control for individual differences.

Strikingly, our methylation data support a potential role of chronic *Ctr* infection as an epigenetic modulator. The molecular mechanisms that lead to the observed increase in differential CpG hypermethylation of genes known to be regulated by polycomb in mouse and human embryonic stem cells^27,37^ are yet to be determined. Yet, it is conceivable that this process is influenced by the transient spike in proliferation during the early phase of *Ctr* infection and the activation of signaling pathways known to maintain crosstalk with EZH2 polycomb networks^38^. Studies of hematopoietic stem cells first reported epigenomic changes that enhance self-renewal in the context of ageing^29^. In this context, it is interesting that we also observed an increase in stemness in chronically infected organoids. It remains to be seen if the shift towards a more de-differentiated phenotype is connected to these epigenetic changes. Notably, though, the number of cilia in non-infected organoids remained constant over time, in congruence with our previous observations^13^—despite the changes in CpG methylation and molecular ageing that were also observed in the non-infected samples. Therefore, these types of epigenetic changes do not appear to be sufficient to alter organoid composition and imply involvement of additional cellular mechanisms triggered by infection.

Taken together, this study highlights progress in the establishment of a next-generation human epithelial in vitro model, enabling investigation of important aspects of the *Ctr*-host interaction. Human FT organoids maintain stemness and differentiation programs—both of which are necessary to allow for detection of the long-term effects of infection on the host epithelium. Such programs are not present in standard monolayer cultures, in particular transformed cell lines. Our findings shed tantalizing new light on the effects of *Ctr* on the tubal epithelium that may have a role in the development of tubal pathology. Although *Ctr* has a relatively high rate of upper genital tract colonization, estimated at ~10% of all infected women, it remains underreported due to the frequent asymptomatic clinical presentation. Given the interference with core developmental mechanisms of epithelial homeostasis, the epigenetic changes and the expression of immune system modifiers we observed, it is clear that future investigation of the long-term consequences of *Ctr* infection is imperative and will need to take into account the interplay between infected epithelium and its tissue microenvironment. This new approach will thus contribute to a better understanding of the long-term effects of *Ctr* infections, including its potential to contribute to cell transformation and the etiology of HGSOC.

## Methods

### Patient material

FT samples were provided by the Department of Gynecology, Charité University Hospital, Campus Virchow Clinic and August-Viktoria Klinikum Berlin, Department of Gynecology and Obstetrics. Scientific usage of the samples for experimental purposes was approved by the Ethics Commission of the Charité, Berlin (EA1/002/07) and informed consent was obtained from all donors. Fragments were sourced from standard surgical procedures for benign gynecological disease. Only anatomically normal FTs were used. Tubes were transported and dissected within 2 to 3 h of removal.

### Organoid cultivation

Generation and culture of FT organoids was performed as described in Kessler et al^13^. In brief, epithelial progenitors from human FT tissue samples were isolated by enzymatic digestion with collagenase I (Sigma). The retrieved primary cells were seeded in 2D culture for 5–7 days (ADF medium with 12 mM HEPES, 1% GlutaMAX^TM^, 10 ng ml^−1^ human EGF and 9 µM Y-27632) before seeding in Matrigel^TM^ (~30,000 cells/50 µl) for 3D organoid formation. Once the Matrigel^TM^ had set, cultures were overlaid with medium containing a specific growth factor cocktail to preserve stemness and support differentiation (ADF, 25% conditioned mouse Wnt3A-medium as described in Willert et al.^39^ and 25% conditioned mouse RSPO1 medium^40^, supplemented with 12 mM HEPES, 1% GlutaMAX^TM^, 2% B27, 1% N2, 10 ng ml^−1^ human EGF (all from Invitrogen), 100  ng ml^−1^ human noggin, 100 ng ml^−1^ human FGF-10 (both from Peprotech), 1 mM nicotinamide, 9 µM ROCK inhibitor (Y-27632, both from Sigma) and 0.5 µM TGF-β RI Kinase Inhibitor IV (SB431542, Calbiochem)). For propagation, organoids were split every 2 to 3 weeks at a ratio of 1:2 to 1:3 using mechanical splitting with a syringe and needle (26 G gauge). Organoids were kept in a humidified incubator at 5% CO_2_ and 37 °C, or 35 °C once infected.

### Organoid formation assay

Single cells were prepared from long-term infected organoids (>2 months p.i.) by digestion with collagenase and reseeded at 40,000 cells/25 µl Matrigel^TM^ in triplicate. At 3 weeks post-seeding, cell viability assay was performed on all wells. The Cell Titer-Glo® 3D Cell Viability kit (Promega # G9681) was applied to each well according to the manufacturer’s protocol and the luminescence measured using a plate reader.

### Antibodies

The following antibodies were used in this study: primary—mouse anti-E-Cadherin (1:200 610181, BD Transduction Lab), mouse anti-β-actin (1:200, A5441, Sigma), goat anti-*Ctr* (OBT0978, AbD Serotec), mouse anti-HSP60 (1:5000, ALX-804-701, Alexis), rabbit anti-Ki67 (9027, Cell Signaling), rabbit anti-pSTAT1 (1:1000, 9167, Cell Signaling), rabbit anti-pSTAT3 (Tyr705) (1:1000 9145, Cell Signaling), cleaved caspase-3 (1:1000, 9664, Cell Signaling), goat anti-LIF (1:500, AF-250-NA, R&D Systems); secondary - sheep anti-mouse-HRP (1:3000, NA931, Amersham), donkey anti-rabbit-HRP (1:3000, NA934, Amersham), donkey anti-goat-HRP (1:3000, 800073, Biomol), donkey anti-mouse-Alexa488 (1:300, 715-546-140, Dianova), donkey anti-goat-Cy3 (1:300, 705-165-003, Dianova), donkey anti-rabbit-Alexa488 (1:300, 711-546-152, Dianova), donkey anti-mouse-Dylight 647 (1:300, 715-605-150, Dianova), CD326 (EpCAM)-FITC antibody (1:50, 130-080-301, Miltenyi), mouse anti-human CD24-BV711 antibody (1:200, 563371, BD Biosciences), mouse anti IgG1-APC (1:100, 130-098-846, Miltenyi), and mouse anti-human CD133/1 (AC133)­APC (1:100, 130­098­829, Miltenyi). As a DNA-labeling agent, Draq5 (5 µM, 62252, Thermo Scientific) was used.

### Single cell preparation and fixation

To prepare single cells, organoids were released from Matrigel using cold PBS, pelleted by centrifugation (5 min, 300 × *g*), resuspended in 0.5–1 ml TrypLE and incubated for 15 min at 37 °C. Organoid fragments were then mechanically disrupted by passing 3–4× through a 26 G gauge needle. Next, the cells were taken up in 3 ml Advanced DMEM/F12 and passed through a 40 µm filter. The single cell suspension was pelleted by centrifugation and resuspended in 500 µl 3.7% PFA for 30 min. Cells were then washed by addition of 3 ml PBS/1% BSA, centrifuged (5 min, 300 × *g*), resuspended in 1 ml PBS/1% BSA and kept at 4 °C for further processing.

### Proliferation assay

To determine the number of proliferating cells, the Click-iT-EdU assay (Thermo Fischer, #C10425) was applied. Organoids were treated with 10 µM EdU for 2 h in culture medium, triturated into single cells and fixed with 3.7% PFA. Labeling of the EdU-positive cells with Alexa488 was done as per manufacturer’s instructions. Flow cytometric analysis was performed using a BD FACS CANTOII flow cytometer and the FlowJo vX.0.6 software.

### Flow cytometry

Single cells derived from organoids were labelled in 1×PBS/1% BSA using mouse anti-human CD326 (EpCAM)-FITC antibody, mouse anti-human CD24-BV711 antibody and mouse anti-human CD133/1 (AC133)­APC. Labeling was performed for 30 min on ice in the dark. Afterwards, cells were washed using 1 ml PBS. Finally, cells were pelleted and resuspended in PBS. The flow cytometric analysis was done using a BD FACS CANTOII flow cytometer and the FlowJo vX.0.6 software.

### Quantitative real-time polymerase chain reaction (qRT-PCR)

Determination of RNA levels was performed by qRT-PCR using the AB Power SYBR^®^ Green RNA-to-CT™ 1-Step Kit (Applied Biosystems^TM^), StepOnePlus™ Real-Time PCR System (Applied Biosystems^TM^), and StepOne^TM^ Software (v2.3, Applied Biosystems).

A mixture of 10 µl RNA (2–5 ng µl^−1^), 10 µl SYBR^®^ green mix, 4.3 µl H_2_O, 0.2 µl Reverse Transcriptase enzyme mix and 0.5 µl Primer mix (10 µM) was subjected to the following PCR cycler program: 30 min 48 °C; 10 min 95 °C; followed by 40× cycles of 15 s 95 °C/60 s 60 °C.

Each sample was measured in triplicate. The expression levels of all target genes were normalized to expression of the housekeeping gene *glyceraldehyde-3-phosphate dehydrogenase* (*GAPDH*). Relative expression levels were determined by calculating ΔΔ*C*_t_. Fold change was calculated as an average of ΔΔ*C*_t_ of independent biological replicates (2−ΔΔ*C*_t_), while s.d. was calculated as ΔΔ*C*_t_ + *s* and ΔΔ*C*_t_ − *s*, where *s* is the pooled s.d. of the Δ*C*_t_ and Δ*C*_t_ control values ($s = \sqrt {{\mathrm{sd}}\left( {{\mathrm{ctt}}} \right)^2 + {\mathrm{sd}}\left( {{\mathrm{ctc}}} \right)^2}$).

The following primers were used: *GAPDH*—forward 5′-GGTATCGTGGAAGGACTCATGAC-3′, reverse 5′-ATGCCAGTGAGCTTCCCGTTCAG-3′; *LIF*—forward 5′-CAGGAGTTGGGTCCAGATGT-3′, reverse 5′-GTCCACAATCTCCCAGAGGA-3′; *Oct4*—forward 5′-GATGTGGTCCGAGTGTGGTTCT-3′, reverse 5′-TTGTGCATAGTCGCTGCTTGA-3′; *CtrD 16S rRNA*—forward 5′-GGTATCGTGGAAGGACTCATGAC-3′, reverse 5′-TCAAATCCAGCGGGTATTAACCGCCT-3′.

### Lentiviral manipulation

Replication-deficient lentiviral particles were produced by transfection of 293T cells with a mixture of the respective lentiviral plasmids and Fugene6 (Promega, #E2691) diluted in Opti-MEM™ (Gibco, # 31985088). For a 10 cm dish, 15.6 µl Fugene6 was mixed with 192.4 µl OPTI-MEM and added to 3 µg lentiviral target plasmid, 2.25 µg psPAX2 packaging vector and 0.75 µg pMD.2 G (VSVG) envelope vector diluted in 52 µl OPTI-MEM. After 30 min incubation at RT, the mixture was pipetted dropwise onto the 293T cells. 16 h post transfection the medium was replaced with 10 ml fresh DMEM. 24 h later the medium was aspirated, filtered at 45 µm and mixed with Lenti-X^TM^ Concentrator (Clontech Laboratories, #631231). After concentration, the virus pellet was resuspended in 1 ml ADF (10x concentration) and stocked at −80 °C.

LIF shRNA lentiviral plasmids and the corresponding vector control were obtained from Sigma (pLKO.5, SHC201; pLKO.5-LIFshRNA: TRCN0000308277, 5′-ACCGCATAGTCGTGTACCTTG-3′; pLKO.1-LIFshRNA: TRCN0000058586, TAAGCAGATCATCGCCGTGTT). The pCT-Mem-GFP plasmid was obtained from System Biosciences (#CYTO100-VA-1).

For transduction, early passage 2D primary FTECs at ~70% confluence in a 6-well plate were treated with 1×, 0.5×, or 0.25× concentrated virus diluted in ADF medium supplemented with 12 mM HEPES, 1% GlutaMAX, 10 ng ml^−1^ human EGF, 9 mM ROCK inhibitor and 5 µg ml^−1^ polybrene (Sigma, #H9268). The cells were incubated overnight with the virus at 37 °C. When the cells reached ~90% confluence, organoids were prepared and selection with puromycin (0.5 µg ml^−1^) was started.

### RNA and DNA isolation

RNA and DNA were isolated using the Allprep Qiagen Kit.

### Microarray RNA isolation, quantification, and quality control

Total RNA was isolated with TRIzol (Life Technologies) according to the supplier’s protocol using glycogen as carrier. Quality control and quantification of total RNA was assessed using an Agilent 2100 Bioanalyzer with an RNA Nano 6000 microfluidics kit (Agilent Technologies) and a NanoDrop 1000 UV-Vis spectrophotometer (Kisker).

### Microarray expression profiling and data analysis

Microarray experiments were performed as dual-color or single-color hybridizations on either 4 × 44 K human catalogue (Agilent-026652) or 8 × 60 K human custom (Agilent-048908) microarrays comprising identical features for coding genes. In order to compensate dye-specific effects and to ensure statistically relevant data, color-swap dye-reversal hybridizations were performed^41^. RNA labeling was done either with a two-color Quick Amp Labeling Kit (4 × 44 K arrays) or with a Low Input Quick Amp Kit (6 × 60 K arrays) according to the supplier’s recommendations (Agilent Technologies). In brief, mRNA was reverse transcribed and amplified using an oligo-dT-T7 promoter primer, and labeled with Cyanine 3-CTP or Cyanine 5-CTP. After precipitation, purification, and quantification, 1 μg (4 × 44 K arrays) or 300 ng (8 × 60 K arrays) of each labeled cRNA was fragmented and hybridized to whole genome multipack microarrays according to the manufacturer’s protocol (Agilent Technologies). Scanning of microarrays was performed with 5 μm resolution and XDR extended range (4 × 44 K arrays) or 3 µm resolution (8 × 60 K arrays) using a G2565CA high-resolution laser microarray scanner (Agilent Technologies). Microarray image data were analyzed and extracted with the Image Analysis/Feature Extraction software G2567AA v. A.11.5.1.1 (Agilent Technologies) using default settings and the protocol GE2_1100_Jul11. The extracted MAGE-ML files were subsequently analyzed with the Rosetta Resolver, Build 7.2.2 SP1.31 (Rosetta Biosoftware). Ratio profiles comprising single hybridizations were combined in an error-weighted fashion to create ratio experiments. A 1.5-fold change expression cut-off for ratio experiments was applied together with anti-correlation of ratio profiles, rendering the microarray analysis highly significant, robust, and reproducible. Additionally, raw data txt files were analyzed with R packages from the Bioconductor repository. The networks and functional analyses of microarray data were generated via Ingenuity Pathway Analysis (QIAGEN Inc., [https://www.qiagenbioinformatics.com/products/ingenuity-pathway-analysis])^42^. The data discussed in this publication have been deposited in NCBI’s Gene Expression Omnibus and are accessible through GEO Series accession number GSE107712.

### GSEA analysis

We derived a stem cell signature using published gene expression data (GSE60919)^13^ from FT organoids upon DBZ treatment. Raw data were normalized using method LOESS and differentially expressed genes determined using R and the BioConductor package LIMMA package^43^. We selected downregulated genes with FDR < 10% and log2 fold change <−1.5 as putative FT stem cell signature genes. We performed GSEA on those genes pre-ranked by gene expression-based *t*-score between infected and non-infected samples at 3 d p.i., using the fgsea R package^44^ with 5000 permutations.

### Live-cell imaging

FT epithelial cells were transduced with a plasmid carrying a GFP-membrane tag (pCT-Mem-GFP plasmid, System Biosciences #CYTO100-VA-1). After selection, GFP mem transduced organoids were infected with mCherry-labeled *Chlamydia* stock according to the standard protocol, seeded into Matrigel in ibidi µ dishes, incubated for 24 h and transferred to an inverted Leica TCS-SP5 confocal microscope equipped with a cell culture incubator. For about 50 h, confocal stacks of about 30 frames were generated every 10 min. These stacks were used for 3D reconstruction and videos using Volocity 6.3.

### Transformation of *Chlamydia* with RFP

The protocol was adapted from procedures developed by Wang and colleagues^45,46^; Agaisse and Derré^47^; Song and colleagues^48^ and Bauler and Hackstadt^49^. In brief, *Chlamydia trachomatis* K (*Ctr* K, a kind gift from Lars Köhler) EBs were transformed with p2TK2-SW2_IncDProm-mCherry-IncDTerm plasmid DNA. The transformed strain was then selected with increasing concentrations of penicillin G for 7 passages until 100 units ml^−1^ for further propagation of transformed *Ctr* K mcherry stock. For the preparation of mCherry *Ctr* K stocks, chlamydial EBs were propagated in HeLa cells (ATCC® CCL-2) in T150 cm^3^ cell culture flasks with 25 ml of culture medium. Cells were infected with lysates from earlier passages and incubated at 35 °C in a cell culture incubator under humidified atmosphere with 5% CO_2_ until time of harvest at 48 h p.i., as described above. The resulting supernatant was collected and centrifuged at 20,000 rcf for 40 min at 4 °C in an SS-34 rotor (Sorval RC 5 C Plus) to pellet chlamydial EBs. The harvested bacteria were resuspended in 5 ml SPG buffer (4 °C). A second centrifugation was performed and the pellet resuspended in 4 ml of SPG buffer (4 °C) and frozen at −80 °C.

### Chlamydia infection, cure, and re-infection of FT organoids

For infection with *CtrD* (ATCC® VR-885TM), *CtrE* (ATCC® VR-348B™) and *CtrK*, 2 wells of organoids were released from Matrigel^TM^ with cold DPBS, pooled and mechanically disrupted by passing them through a 26 gauge needle. The fragmented organoids were divided evenly into 2 tubes for infection and mock control. After centrifugation (300 × *g*, 5 min) and removal of the supernatant, 10 µl *Ctr*D (from a frozen stock with 10^8^ IFU ml^−1^ as determined by titration on HeLa cells) were added to one tube with the pelleted organoids. The suspension was mixed by pipetting and incubated for 15 min at 35 °C followed by 5 min on ice. The mock control was treated in the same way, but without addition of *Ctr*D. Finally, 50 µl Matrigel^TM^ was added to each tube, the organoids seeded and placed in a humidified incubator at 5% CO_2_ and 35 °C. Splitting of organoids was done every 3–4 weeks. RNA samples were taken at 72 h p.i. and 1 month p.i.

At around 4 months p.i. the infection was cured by applying a mixture of penicillin (100 U ml^−1^) and streptomycin (100 µg ml^−1^) to the organoid culture for one week. After curing, re-infection was carried out in the same way as the first infection with mechanical splitting, adding of *Ctr*D to one half of the organoids and mock treatment of the other half. Control organoids initially infected with *Ctr*D were infected or mock-treated again. 72 h post seeding, RNA samples were taken of the now 2× infected (chronic infected + re-infected), chronic infected (chronic cured), acute infected (long-term acute inf), and the never infected organoids (Fig. 3d).

### RNA samples for microarray

At the respective time points (3 d p.i., 1 month p.i. and 4 months p.i.) organoids were released from Matrigel^TM^ with ice-cold DPBS and centrifuged (300 × *g*, 5 min). The cell pellet from one Matrigel^TM^ drop was resuspended in 750 µl TRIzol^®^ reagent (Invitrogen # 15596026) and stored overnight at −80 °C until further isolation.

### Infectivity assay

One day before the infectivity assay, HeLa cells were seeded at a density to achieve ~ 70% confluence at the time of infection. Both *Ctr*-infected and non-infected FT organoids were treated as follows: Medium was removed and Matrigel^TM^ dissolved in 1 ml ice-cold RPMI w/o FCS. Organoids were centrifuged for 4 min at 300 × *g*. After removal of the supernatant, cells were resuspended in 1 ml RPMI and homogenized by passing three times through a 26 G gauge needle. After another round of centrifugation and addition of 1 ml ice-cold RPMI, the pellet was transferred to a 50 ml falcon tube with 1 ml sterile glass beads. EBs were released by vigorous vortexing for 5 min. After centrifugation for 4 min at 300 × *g*, the supernatants were transferred to the prepared HeLa cells containing fresh medium. After 24 h (at 35 °C, 5% CO_2_) cells were fixed in PFA and subjected to IF staining for detection of inclusions.

### Immunofluorescence staining

Organoids were fixed with 3.7% PFA, embedded in paraffin, sectioned and stained for confocal imaging as described previously^13^.

### Whole-mount staining

Organoids were released from Matrigel with 1xPBS and pelleted by gravitational flow to maintain 3D structure. After fixing with 3.7% PFA for 1 h at RT, organoids were permeabilized and blocked overnight at 4 °C in 5% donkey serum, 1% FCS, 0.05% Tween-20, 0.5% Triton X-100 and 0.02% sodium azide in 1×PBS. Staining with primary antibodies was performed in blocking buffer (0.0025% Triton X-100) at a dilution of 1:200 for 3 days at 4 °C. Washing (5 × 45 min in 1×PBS with 5% glycerol) was followed by staining with Draq5 and secondary antibody in blocking solution (0.0025% Triton X-100) at a dilution of 1:200 for 2 days at 4 °C. After washing, organoids were transferred onto an ibiTreat µ-slide (ibidi #003031) and *z* stacks performed using confocal microscopy. The 3D structure was reconstructed and visualized using Fiji (ImageJ).

### Western blot

Organoid samples were treated with ice-cold PBS to remove Matrigel, centrifuged (300 × *g*; 5 min; 4 °C), supernatant was removed and the cell pellet resuspended in 100 µl SDS sample buffer (4% SDS, 32% glycerol, 125 mM Tris pH 6.8, 200 mM β-mercaptoethanol, and bromphenol blue). Samples were boiled for 7 min at 96 °C, then separated via SDS-Page electrophoresis (~90 min at 150 V const) and transferred to a PVDF membrane (2.5 h at 250 mA const. at 4 °C). Membranes were blocked in 5% skim milk/TBS-Tween-20 (0.1%) for 60 min. Primary antibodies were diluted 1:500 or 1:1000 in 5% skim milk/TBS-Tween-20 (0.1%) and incubated with the blocked membranes at 4 °C overnight. After extensive washing in TBS-T for 45 min in total, secondary antibodies anti-mouse- or anti-rabbit-HRP were diluted 1:3000 in 5% skim milk/TBS-Tween-20 (0.1%) and incubated for 1 h at room temperature. ECL detection was performed according to the manufacturer’s protocol after washing the membranes with TBS-T for 1 h. Uncropped scan images of all blots are provided in Supplementary Figure 9.

### LIF neutralization

For LIF neutralization the general protocol for *CtrD* infection of organoids (as described above) was carried out. Differing from this protocol, the fragmented organoids were resuspended in 90 µl ADF++, which was added with or without *CtrD*, and subsequently mixed with 10 µl of 1:40 pre-diluted antibodies. Neutralization was done using 0.5 µg ml^−1^ anti-LIF antibody (R&D, #AF-250-NA, from goat, 200 µg ml^−1^) and mock control using 0.5 µg ml^−1^ goat anti-CagA (Santa Cruz, #sc-6085, 200 µg ml^−1^). After the specified incubation time for infection, the organoids were seeded in Matrigel and culture medium was added. 14 days p.i. organoids were fixed, embedded and IF labeled for detection of *Chlamydia*.

### Methylation array and bioinformatic analysis

For genome-wide DNA methylation analysis, bisulfite conversion of the genomic DNA and hybridization to the Illumina Infinium® MethylationEPIC BeadChip were performed at Life&Brain (Bonn, Germany), according to the manufacturer’s protocol. Data analysis was carried out in the R environment^50^. Preprocessing and quality control (QC) were performed following the ChAMP package^51^ default filtering steps (probes with a detection *p*-value > 0.01 and with a bead count < 3 were excluded), as well as probes where the sequence overlaps single-nucleotide polymorphisms (SNPs) or that were shown to cross-hybridize according to Zhou et al.^52^ Subset-quantile within array normalization (SWAN) was applied in order to adjust for the type I and type II bias of the methylation array^53^. Differentially methylated CpGs between infected (*Ctr*D) and non-infected (NI) organoids were determined using limma^43^ and a moderated paired *t*-test. A false discovery rate (FDR) was computed by adjusting *p*-values for multiple testing using the Benjamini–Hochberg procedure. For patient-specific DM CpGs upon infection and long-term culture, a cut-off of delta beta > 20% was applied and justified with data from a control experiment (Supplementary Figure 8a,b). Unique DM CpGs compared to the baseline were subjected to a locus overlap analysis (LOLA)^26^ using the databases ENCODE transcription factor binding sites (TFBSs), ENCODE segmentation^37^, UCSC features^54^ as well as Roadmap Epigenomics^55^. For the *χ*^2^ test, for each donor the frequency of hypomethylated or unchanged CpGs in each arm was compared with the corresponding number of hypermethylated CpGs. The Methylation BeadChip data generated in this study have been deposited in the National Centre for Biotechnology Information Omnibus (GEO) under the accession code GSE108202.

### Reporting summary

Further information on experimental design is available in the Nature Research Reporting Summary linked to this article.

## Supplementary information

Supplementary Information

Description of Additional Supplementary Files

Supplementary Data 1

Supplementary Data 2

Supplementary Movie 1

Reporting Summary

## Data Availability

The microarray and methylation BeadChip data from this manuscript have been deposited in the National Centre for Biotechnology Information Omnibus (GEO) under accession codes GSE107712 and GSE108202. Raw data associated with Figs. 3f and 4 can be found in Supplementary Data 1 and 2, respectively. Other data supporting the findings of this study are available within the paper and its Supplementary Information files, or from the corresponding author upon request. As far as ethical and legal constrains permit, all biological materials are available upon request from the corresponding author.
