# Supplementary material for: Chronic Chlamydia infection in human organoids increases stemness and promotes age-dependent CpG methylation
Source: Nat Commun. 2019 Mar 18;10:1194. doi: 10.1038/s41467-019-09144-7 (PMC6423033; doi:10.1038/s41467-019-09144-7)
Supplement: Supplementary file 1 — Supplementary Information [file 41467_2019_9144_MOESM1_ESM.pdf]

Supplementary Information

Chronic *Chlamydia* infection in human organoids increases stemness and promotes age-dependent CpG methylation

Kessler M et al

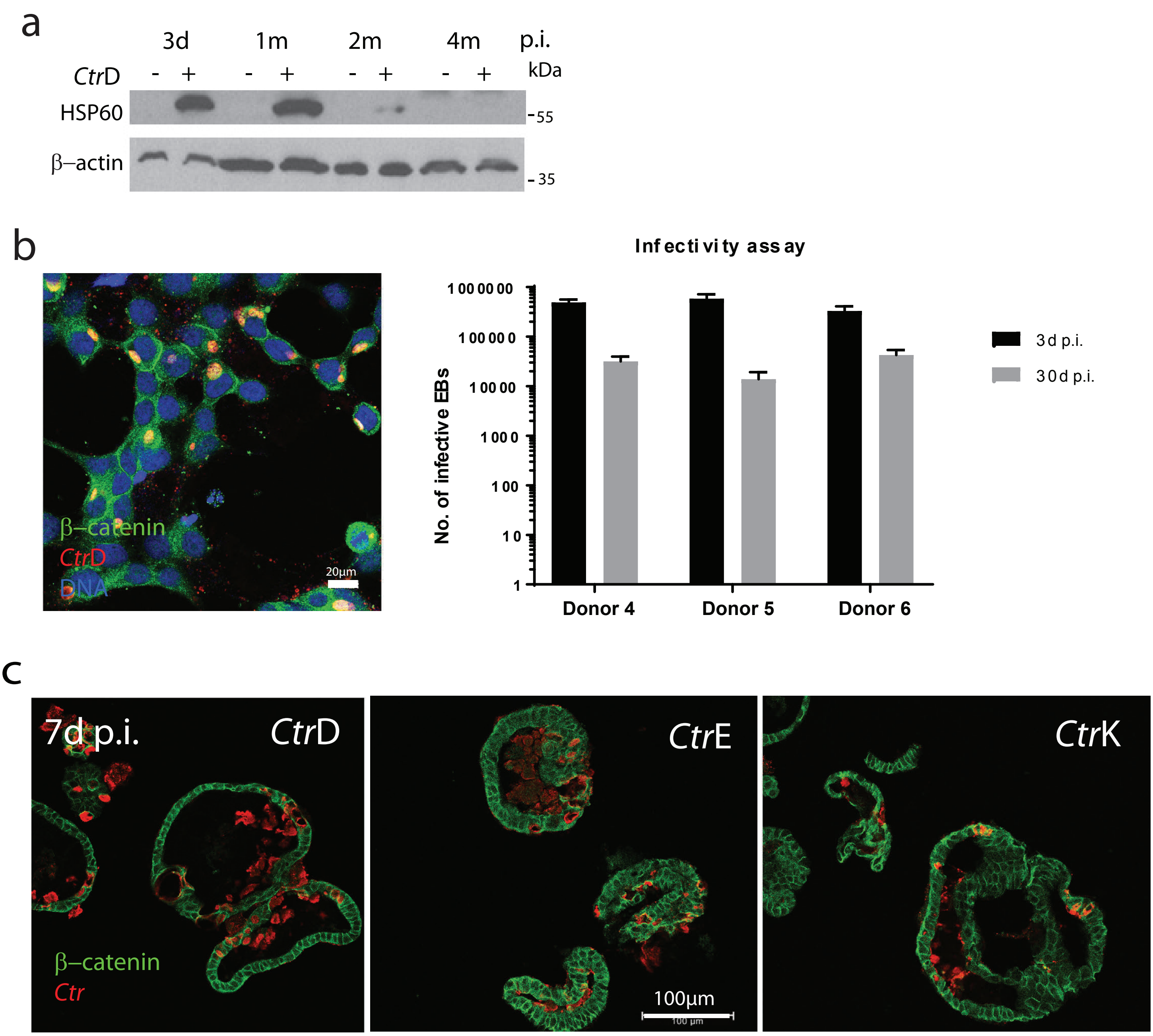

Supplementary Figure 1. Chronic, productive *Ctr* infections in organoids gradually decreases in titer a) Representative blot of HSP60 protein levels from one chronically infected culture at 3 d, 1 month and 4 month p.i. While bacterial load is high at 3 dp.i and remains substantial at 1 month p.i. it decreases to undetectable levels by 4 months p.i. b) Confocal image of HeLa cells containing inclusions filled with *Ctr*, confirming infectious potential of EBs retrieved from infected organoids. The graph represents quantification from 3 independent chronic infections in 3 donors at 3 d and 1 m p.i. ± sd calculated based on inclusion counts from different fields of view. c) Confocal images of representative organoids infected with serovars D, E and K at 7 d p.i. reveals similar infection rates and accumulation of bacteria shed in the organoid lumen.

Interferon signaling activation in acute *Ctr*D infection

a

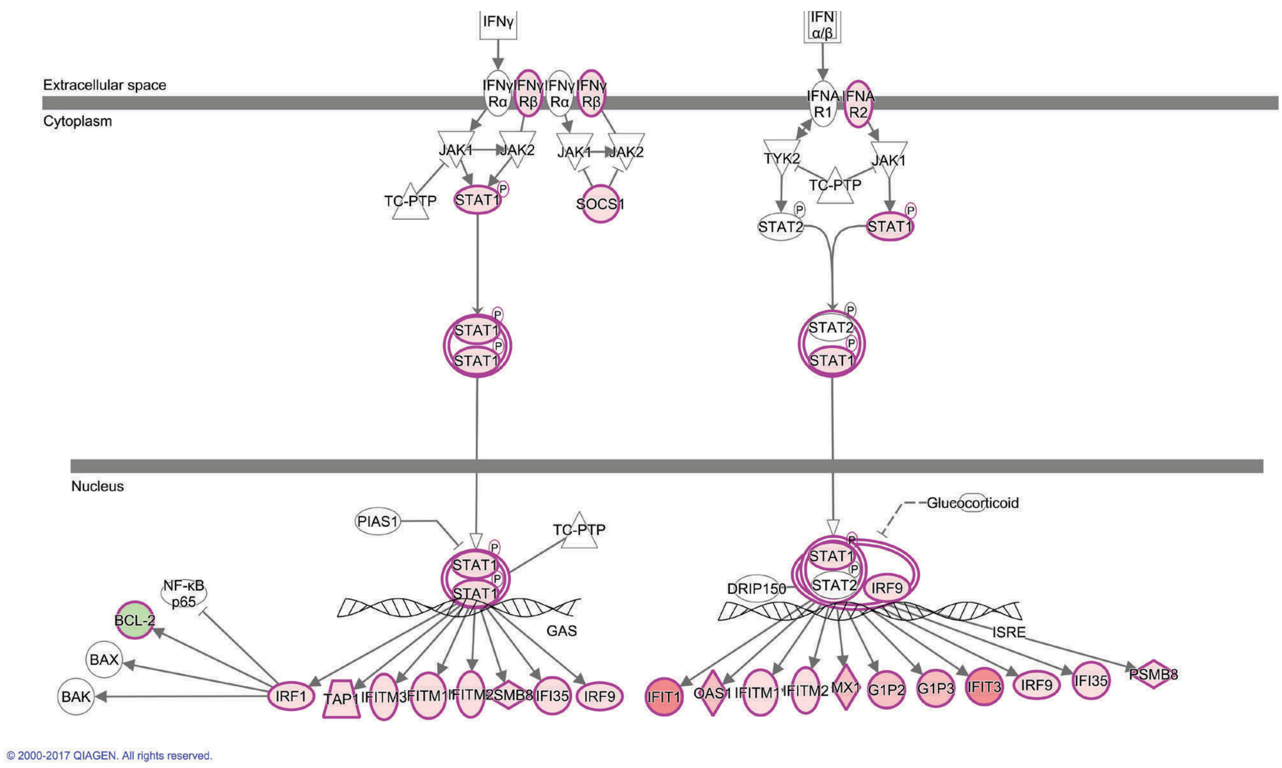

iNOS signaling activation

b

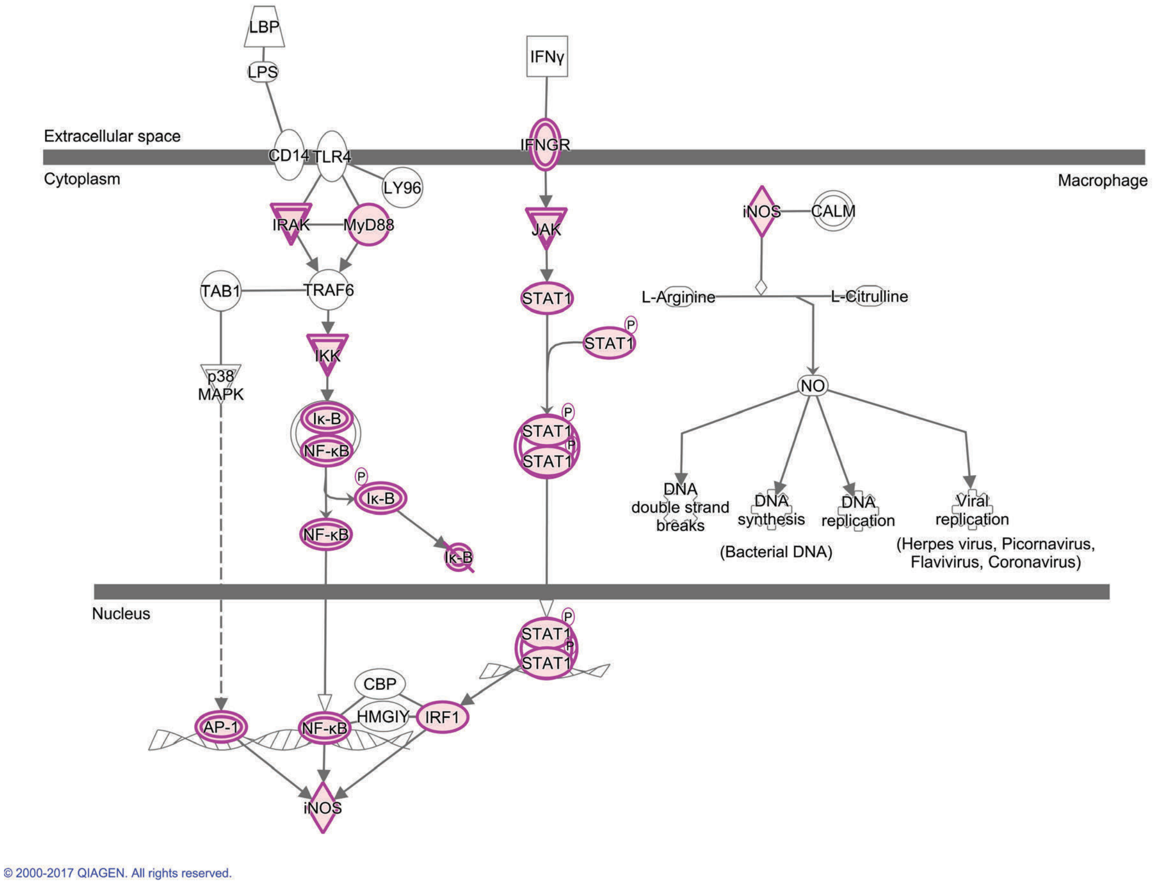

Supplementary Figure 2. Acute *Ctr* infection triggers strong upregulation of interferon response a) Genes in the interferon signaling network that were upregulated (red) during acute *Ctr* infection. b) Genes in the iNOS pathway upregulated during acute *Ctr* infection. Pathways were generated on the IPA^{tm} platform by overlay of canonical networks with microarray data of genes that are regulated in an identical fashion in all 3 donors.

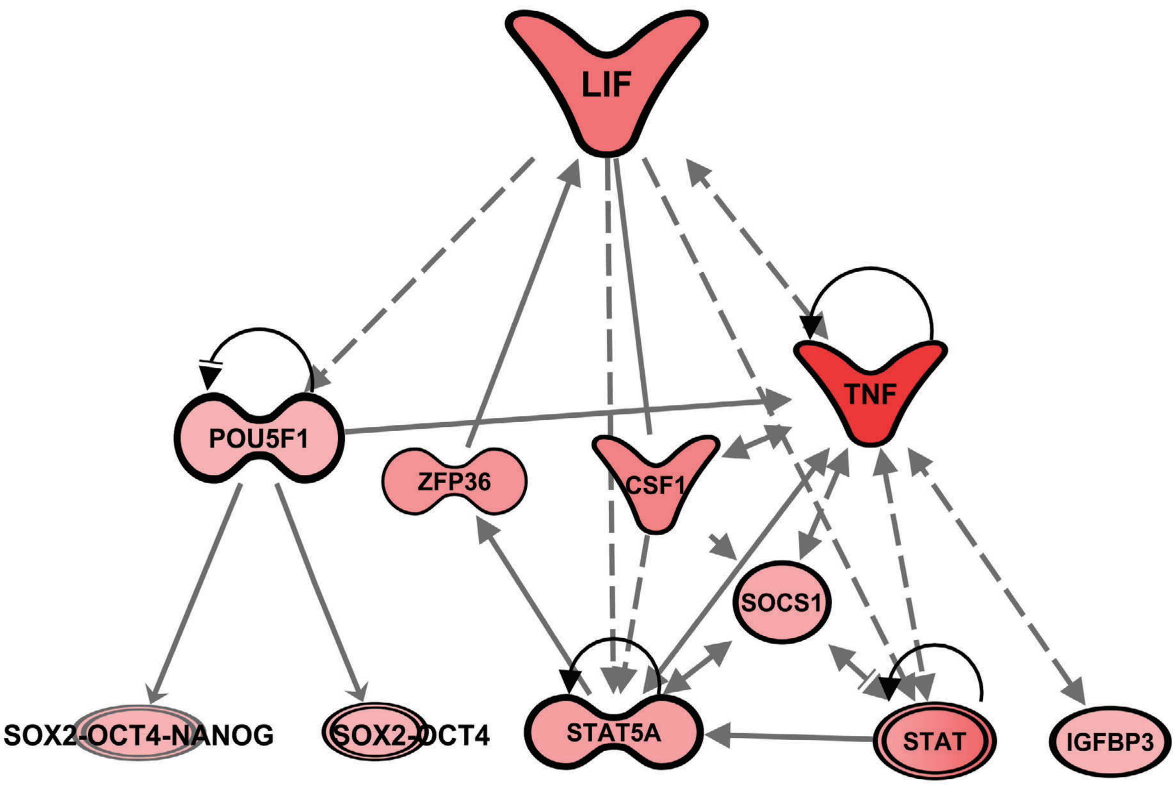

Supplementary Figure 3. Activation of the LIF pathway occurs during acute *Ctr* infection Graphical presentation of the strong upregulation of the LIF gene and associated interactors (all in red). The network was generated on the IPA^{tm} platform by pathway analysis of microarray data of all candidate genes which were consistently regulated in 3 different donors.

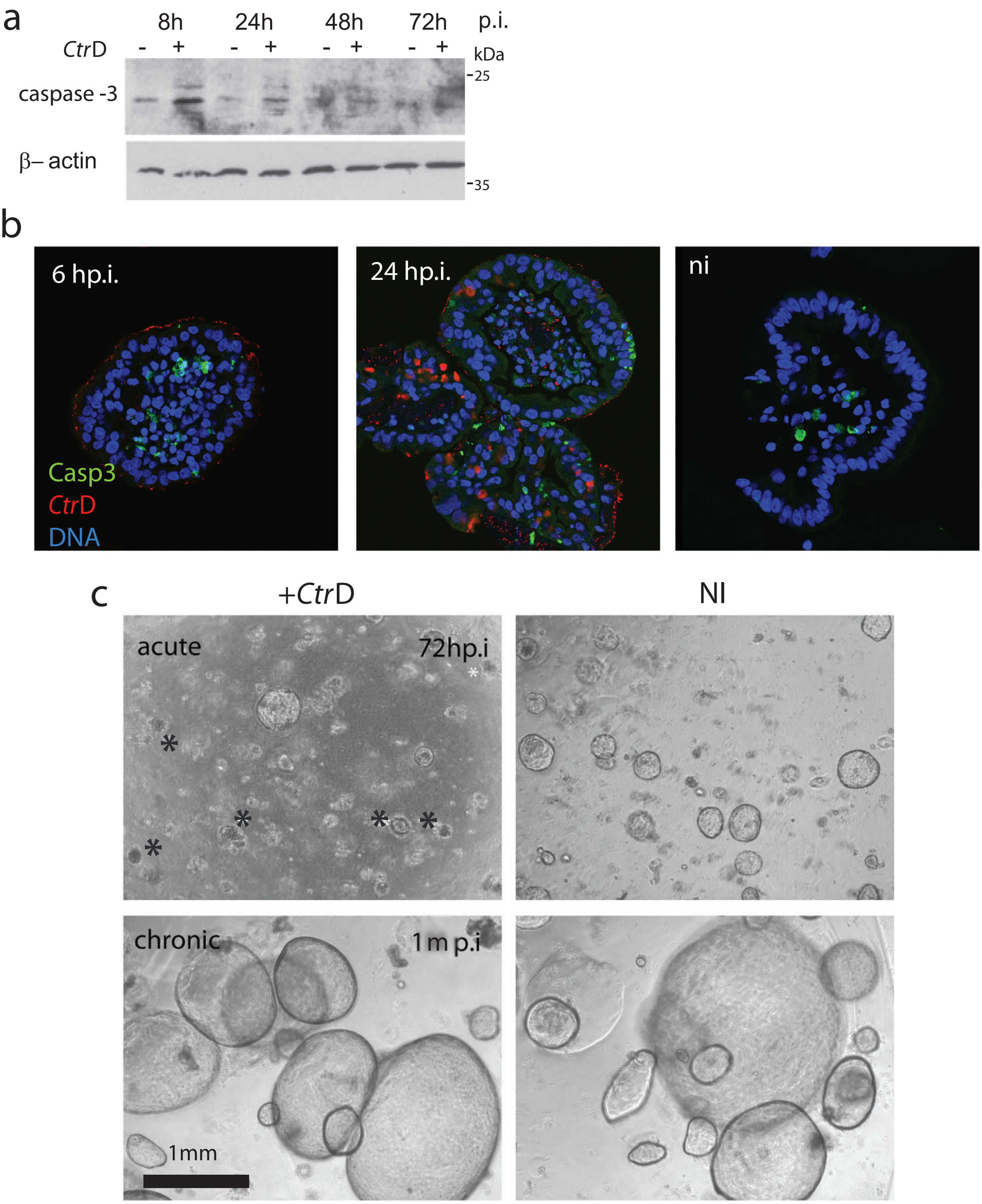

Supplementary Figure 4. Early stress response and apoptosis are compensated during chronic infection a) Western blot for active, cleaved caspase-3 showing a peak in apoptosis in infected organoids at early stages of infection (from 8 -72 h, with decreasing intensity). b) Confocal images of active caspase-3 positive cells at 6 and 24 h p.i. shows an absence of colocalization with inclusion-harboring cells *(Ctr*, red) suggesting a broader stress response and paracrine induction of apoptosis. c) While organoid culture at 72 h p.i. shows clear signs of stress (*) in comparison to control organoids, infected and non–infected cultures at 1 m p.i. have a highly similar phenotype as visible in phase contrast images.

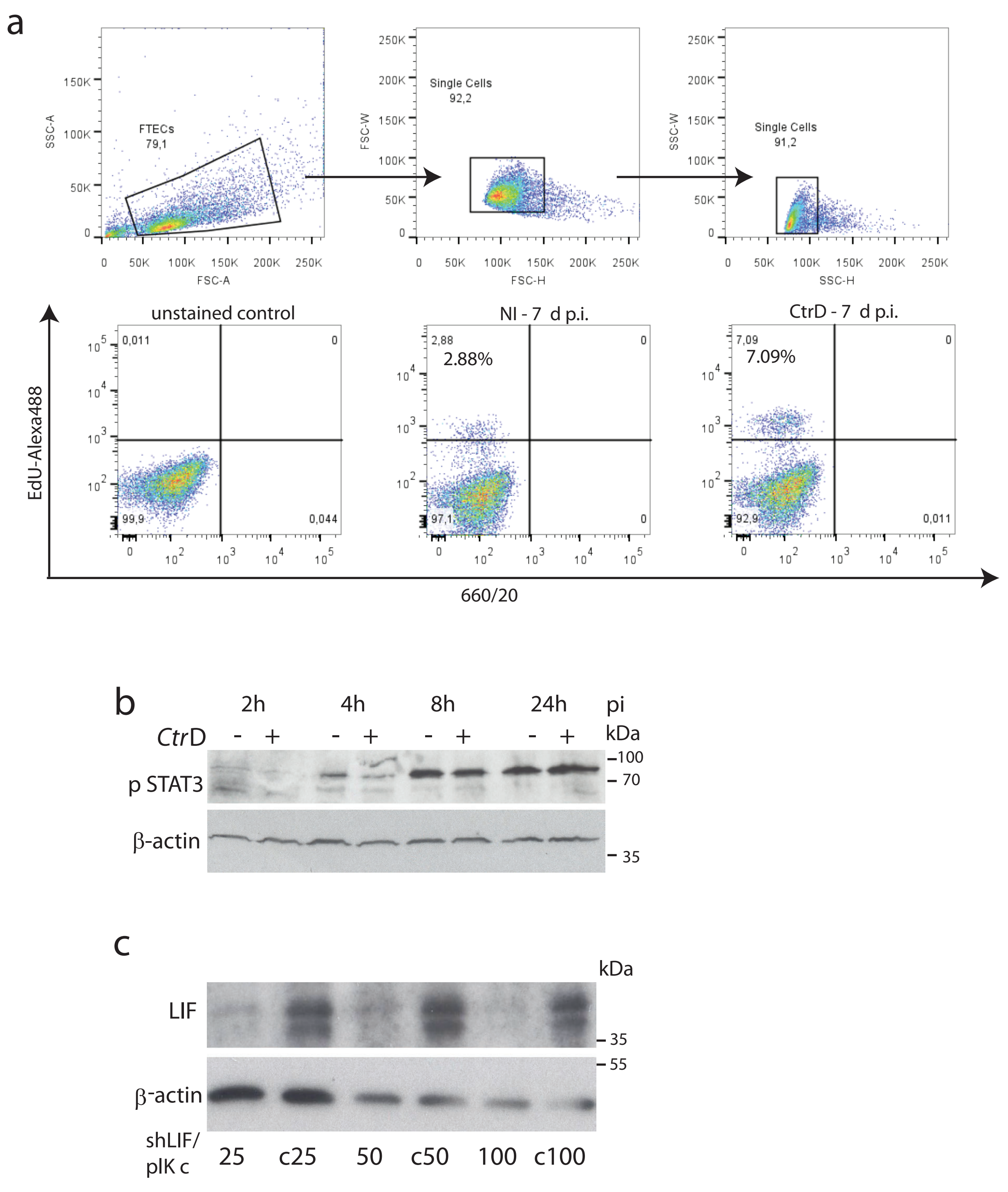

Supplementary Figure 5. LIF signaling is active in organoids independent of presence of *Ctr* a) Gating strategy to count Alexa 488+ (EdU+ proliferative cells) in infected and non-infected organoids over the course of 1 month. b) WB of the time course experiment showing that the pSTAT3 signal transiently disappears after the dissociation of organoids but recovers in both infected and non-infected cultures within a few hours after reseeding c) Depletion of LIF protein by introduction of shLIF is comparable at different virus concentrations, suggesting that a basal level of LIF is required for organoid growth.

a

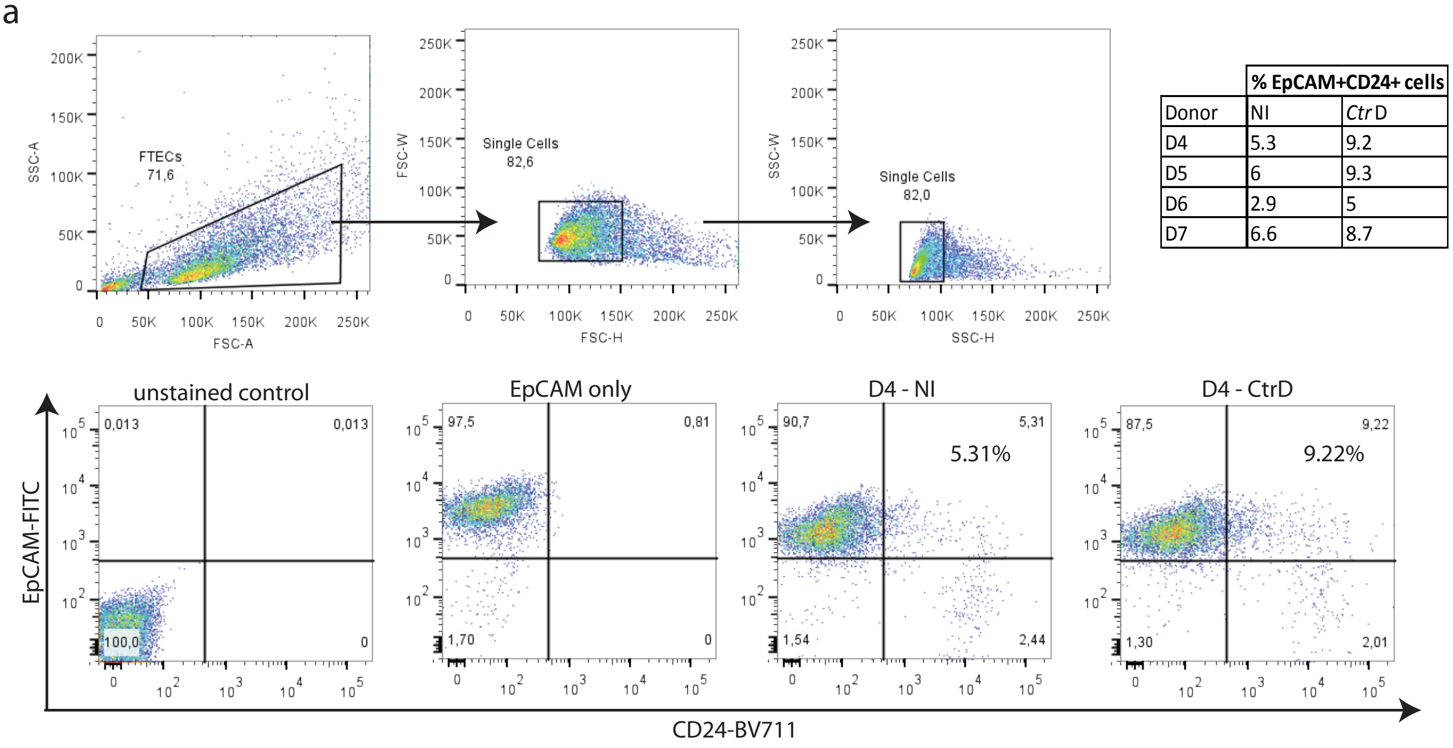

	% EpCAM+CD24+ cells	
Donor	NI	*Ctr* D
D4	5.3	9.2
D5	6	9.3
D6	2.9	5
D7	6.6	8.7

b

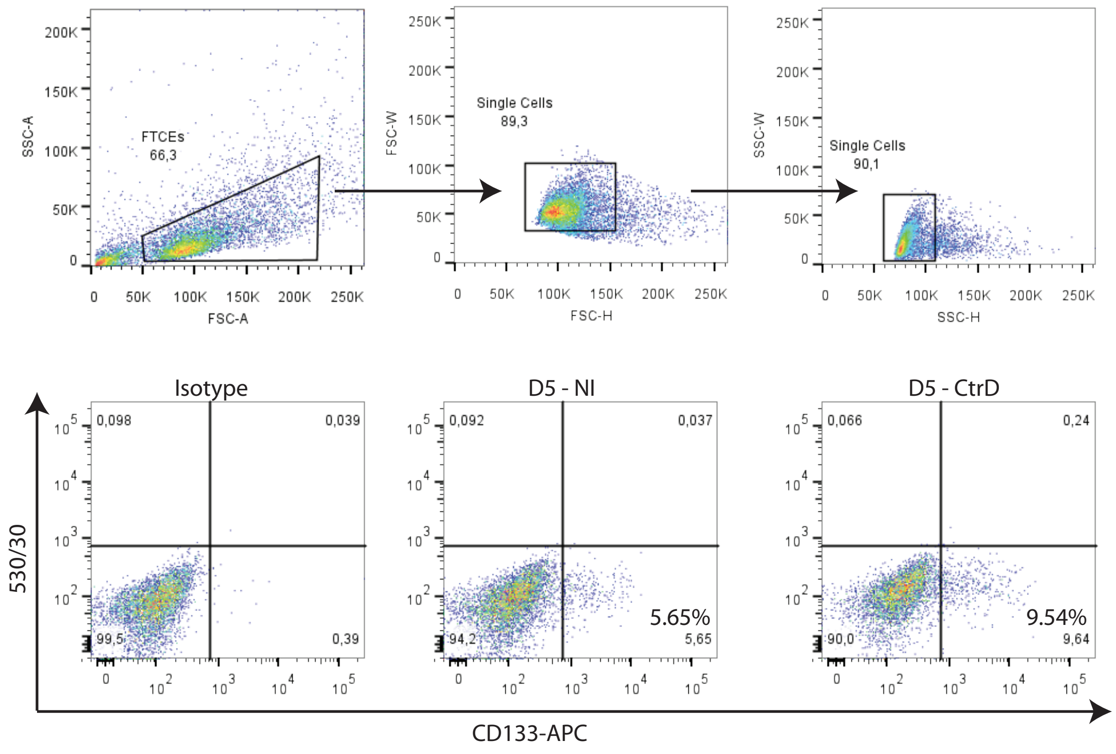

	% CD133+ cells	
Donor	NI	*Ctr*D
D4	1.0	3.7
D5	5.6	9.5
D6	5.0	5.2

Supplementary Figure 6. Chronically infected organoids show an increase in the CD24 and CD133 positive cell populations. a) Chronically infected organoids show an increase in the number of CD24+/EpCam+ cells. Depicted are representative plots from one of four independent FACS sorting experiments. b) Number of CD133+ cells acquired by FACS analysis of non-infected and infected organoid samples for three independent donors. Two out of three cultures infected with *Ctr*D for 2 months demonstrate a clear increase in the size of the CD133+ population.

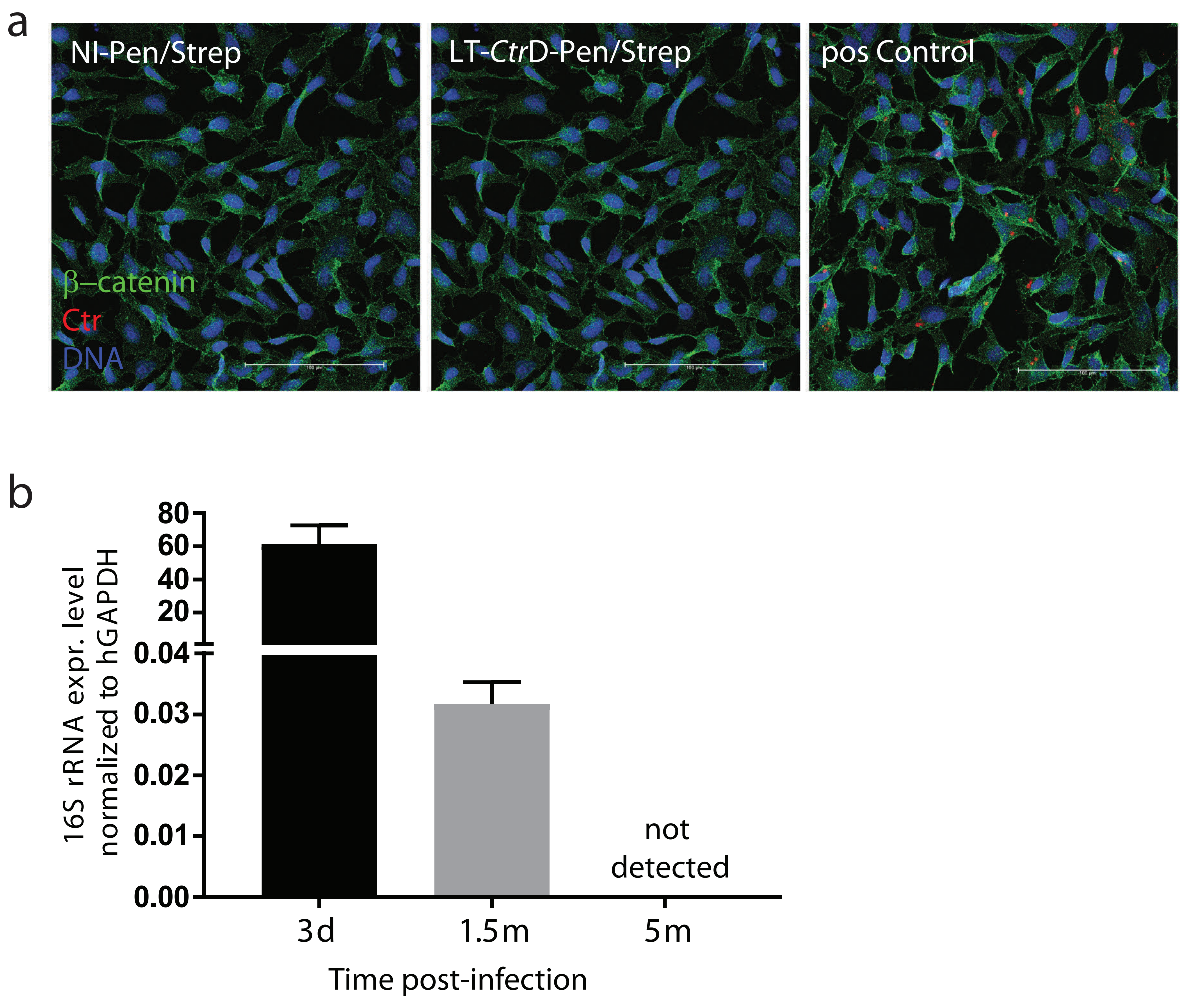

Supplementary Figure 7. Chronically infected organoids show a significantly decreased bacterial load. a) Immunofluorescence labeling of infectivity assay in Hela cells with lysate from long-term infected (LT-*Ctr*D) as well as non-infected (NI) organoids treated for 7 days with Pen/Strep. While the positive control (from 96 hp.i.) contains many inclusions, the absence of inclusions in the NI and P/S –treated chronically infected samples confirms that there were no replicative bacteria left in the culture. b) The relative expression level of bacterial 16S ribosomal RNA was determined by qPCR at different time points of the infected organoid culture and normalized to human GAPDH expression. Data is presented as mean ± sd. At 5 months p.i. the RNA content was below detection level.

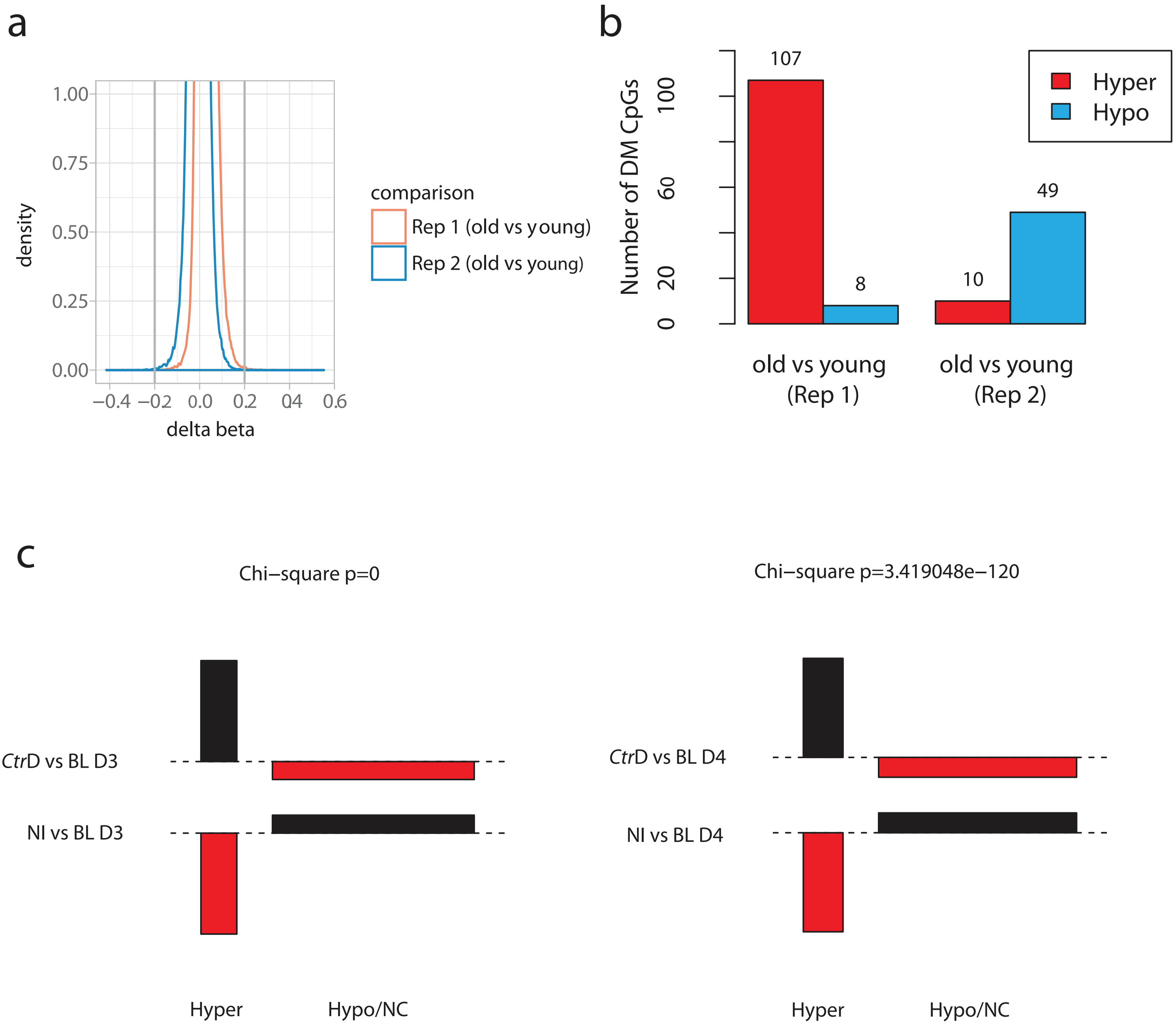

Supplementary Figure 8. Statistical analysis of methylation data in Fig. 4. a) Density plot (zoomed) of the delta beta distribution from two different donors (Rep 1 and Rep 2) comparing two different types of short-term culture. Grey vertical lines indicate the cut off of |delta beta| > 0.2. b) Barplot showing the number of differentially methylated CpGs that pass the threshold determine in a. c) Association plot of the chi-squared test applied to Fig. 4a showing the Pearson residuals ((observed-expected)/sqrt(expected)) in the height of the boxes and the expected values as width.

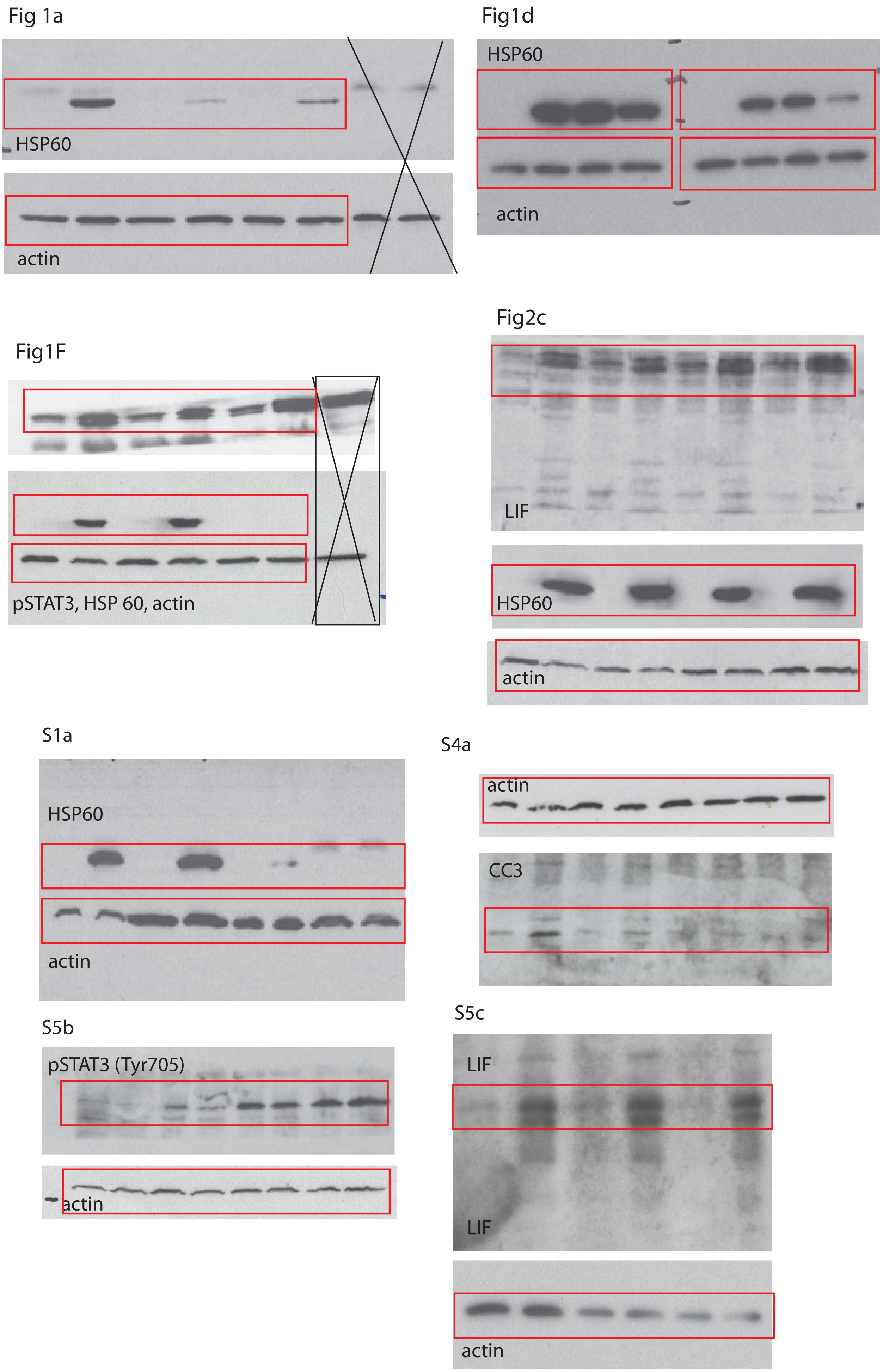

Supplementary Figure 9. Uncropped scans of blots presented in the manuscript. Cropped areas are indicated by red boxes.
