# Supplementary material for: Chronic Chlamydia infection in human organoids increases stemness and promotes age-dependent CpG methylation
Source: Nat Commun. 2019 Mar 18;10:1194. doi: 10.1038/s41467-019-09144-7 (PMC6423033; doi:10.1038/s41467-019-09144-7)
Supplement: Supplementary file 2 — Description of Additional Supplementary Files [file 41467_2019_9144_MOESM2_ESM.pdf]

Description of Additional Supplementary Files

File Name: Supplementary Data 1
Description: List of 91 genes that were significantly differentially regulated across 3 donors after curing of chronic infection.

File Name: Supplementary Data 2
Description: List of 603 CpGs that were differentially methylated across 3 different donors.

File Name: Supplementary Movie 1
Description: Live cell imaging of infected organoid showing dynamic growth of *Ctr* inclusions within an epithelial monolayer. Notably, inclusions are being expelled into the lumen, after which they burst.
