# Supplementary material for: Chronic Chlamydia infection in human organoids increases stemness and promotes age-dependent CpG methylation
Source: Nat Commun. 2019 Mar 18;10:1194. doi: 10.1038/s41467-019-09144-7 (PMC6423033; doi:10.1038/s41467-019-09144-7)
Supplement: Supplementary file 6 — Reporting Summary [file 41467_2019_9144_MOESM6_ESM.pdf]

nature research

Corresponding author(s): Thomas F Meyer

Last updated by author(s): Feb 18, 2019

Reporting Summary

Nature Research wishes to improve the reproducibility of the work that we publish. This form provides structure for consistency and transparency in reporting. For further information on Nature Research policies, see Authors & Referees and the Editorial Policy Checklist.

Statistics

For all statistical analyses, confirm that the following items are present in the figure legend, table legend, main text, or Methods section.

n/a	Confirmed	
☐	☒	The exact sample size (*n*) for each experimental group/condition, given as a discrete number and unit of measurement
☐	☒	A statement on whether measurements were taken from distinct samples or whether the same sample was measured repeatedly
☐	☒	The statistical test(s) used AND whether they are one- or two-sided<br>*Only common tests should be described solely by name; describe more complex techniques in the Methods section.*
☐	☒	A description of all covariates tested
☐	☒	A description of any assumptions or corrections, such as tests of normality and adjustment for multiple comparisons
☐	☒	A full description of the statistical parameters including central tendency (e.g. means) or other basic estimates (e.g. regression coefficient) AND variation (e.g. standard deviation) or associated estimates of uncertainty (e.g. confidence intervals)
☐	☒	For null hypothesis testing, the test statistic (e.g. *F*, *t*, *r*) with confidence intervals, effect sizes, degrees of freedom and *P* value noted<br>*Give P values as exact values whenever suitable.*
☒	☐	For Bayesian analysis, information on the choice of priors and Markov chain Monte Carlo settings
☒	☐	For hierarchical and complex designs, identification of the appropriate level for tests and full reporting of outcomes
☒	☐	Estimates of effect sizes (e.g. Cohen's *d*, Pearson's *r*), indicating how they were calculated

Our web collection on statistics for biologists contains articles on many of the points above.

Software and code

Policy information about availability of computer code

Data collection	*Provide a description of all commercial, open source and custom code used to collect the data in this study, specifying the version used OR state that no software was used.*
Data analysis	fgsea R package (Sergushichev, A. An algorithm for fast preranked gene set enrichment analysis using cumulative statistic calculation. bioRxiv doi: 10.1101/060012(2016).)<br>ChAMP package (Morris, T.J., et al. ChAMP: 450k Chip Analysis Methylation Pipeline. Bioinformatics 30, 428-430 (2014).)<br>limma (Ritchie, M.E., et al. limma powers differential expression analyses for RNA-sequencing and microarray studies. Nucleic Acids Res. 43, e47 (2015))<br>The networks and functional analyses of microarray data (igure 1 f , g, and S2 a , b and S3) were generated through the use of IPA (QIAGEN Inc., https://www.qiagenbioinformatics.com/products/ingenuity-pathway-analysis; Causal analysis approaches in Ingenuity Pathway Analysis. Bioinformatics. 2014 Feb 15;30(4):523-30.

For manuscripts utilizing custom algorithms or software that are central to the research but not yet described in published literature, software must be made available to editors/reviewers. We strongly encourage code deposition in a community repository (e.g. GitHub). See the Nature Research guidelines for submitting code & software for further information.

Data

Policy information about availability of data

All manuscripts must include a data availability statement. This statement should provide the following information, where applicable:

- Accession codes, unique identifiers, or web links for publicly available datasets
- A list of figures that have associated raw data
- A description of any restrictions on data availability

The microarray and methylation BeadChip data from this manuscript have been deposited in the National Centre for Biotechnology Information Omnibus (GEO)

under accession codes GSE107712 and GSE108202.
Raw data associated with Fig. 3f and Fig 4 can be found in Supplementary Tables 1 and 2, respectively.
Quantitative data supporting the findings of this study are available within the paper and its supplementary information files. All other data supporting these findings are available from the corresponding author upon reasonable request.

Field-specific reporting

Please select the one below that is the best fit for your research. If you are not sure, read the appropriate sections before making your selection.

☒ Life sciences ☐ Behavioural & social sciences ☐ Ecological, evolutionary & environmental sciences

For a reference copy of the document with all sections, see nature.com/documents/nr-reporting-summary-flat.pdf

Life sciences study design

All studies must disclose on these points even when the disclosure is negative.

Sample size	No statistical methods were used to predetermine sample size
Data exclusions	No data were excluded from the experiments
Replication	All attempts at replication were successful. All graphs represent data with at least two biological replicates, all images represent findings reproduced at least twice in the laboratory
Randomization	No specific procedures were carried out for randomization
Blinding	The investigator was blinded for image and quantitative analysis

Reporting for specific materials, systems and methods

We require information from authors about some types of materials, experimental systems and methods used in many studies. Here, indicate whether each material, system or method listed is relevant to your study. If you are not sure if a list item applies to your research, read the appropriate section before selecting a response.

Materials & experimental systems

n/a	Involved in the study	
☐	☒	Antibodies
☐	☒	Eukaryotic cell lines
☒	☐	Palaeontology
☒	☐	Animals and other organisms
☐	☒	Human research participants
☒	☐	Clinical data

Methods

n/a	Involved in the study	
☒	☐	ChIP-seq
☐	☒	Flow cytometry
☒	☐	MRI-based neuroimaging

Antibodies

Antibodies used	mouse anti-E-Cadherin (1:200 610181, BD Transduction Lab), mouse anti-β-actin (1:200, A5441, Sigma), goat anti-Ctr (OBT0978, AbD Serotec), mouse anti-HSP60 (1:5000, ALX-804-701, Alexis), rabbit anti-Ki67 (9027, Cell Signaling), rabbit anti-pSTAT1 (1:1000, 9167, Cell Signaling), rabbit anti-pSTAT3 (Tyr705) (1:1000 9145, Cell Signaling), cleaved caspase-3 (1:1000, 9664, Cell Signaling), goat anti-LIF (1:500, AF-250-NA, R&D Systems),sheep anti-mouse-HRP (1:3000, NA931, Amersham), donkey anti-rabbit-HRP (1:3000, NA934, Amersham), donkey anti-goat-HRP (1:3000, 800073, Biomol), donkey anti-mouse-Alexa 488 (1:300, 715-546-140, Dianova), donkey anti-goat-Cy3 (1:300, 705-165-003, Dianova), donkey anti-rabbit-Alexa 488 (1:300, 711-546-152, Dianova), donkey anti-mouse-Dylight 647 (1:300, 715-605-150, Dianova), CD326 (EpCAM)-FITC (1:50, 130-080-301, Miltenyi), mouse anti-human CD24-BV711 (1:200, 563371, BD Biosciences), mouse anti IgG1-APC (1:100, 130-098-846, Miltenyi) and mouse anti-human CD133/1 (AC133)-APC (1:100, 130-098-829, Miltenyi).
Validation	Validation was performed by the manufacturers; positive and negative controls were used in the lab for validation of each antibody

Eukaryotic cell lines

Policy information about cell lines

Cell line source(s)	HeLa cells, ATCC® CCL-2

Authentication	HeLa cells were ordered as an authenticated cell line from ATCC
Mycoplasma contamination	All cell lines tested negative for mycoplasma contamination.
Commonly misidentified lines (See ICLAC register)	We used HeLa cells for Chlamydia stock expansion and infectivity assay as they are the standard cell line used for confirming infection with Chlamydia trachomatis.

Human research participants

Policy information about studies involving human research participants

Population characteristics	Tissue was donated by female patients aged 40 to 75
Recruitment	2014-2018
Ethics oversight	Ethics comimssion Charite Medical University Berlin

Note that full information on the approval of the study protocol must also be provided in the manuscript.

Flow Cytometry

Plots

Confirm that:

☒ The axis labels state the marker and fluorochrome used (e.g. CD4-FITC).

☒ The axis scales are clearly visible. Include numbers along axes only for bottom left plot of group (a 'group' is an analysis of identical markers).

☒ All plots are contour plots with outliers or pseudocolor plots.

☒ A numerical value for number of cells or percentage (with statistics) is provided.

Methodology

Sample preparation	CtrD infected as well as non-infected fallopian tube organoids were split at indicated time points into single cells by enzymatic (TrypLE, 15 min, 37°C) and mechanical (needle, 26G) treatment. Single cell suspensions were filtered (0.45 µm), fixed in 3.7% PFA (30 min, RT) and washed with 1%BSA/PBS. Fixed cells were stained in 1%BSA/PBS with either CD24-BV711 and EpCAM-FITC or CD133-APC (30 min on ice). After staining, cells were washed with PBS and analysed using a flow cytometer. For assessing the number of proliferating cells a Click-iT-EdU assay (C10425, Thermo Fisher) was performed. For this organoids were incubated for 2 h with 10 µM EdU, followed by single cell preparation and fixation. Permeabilization and labeling of EdU positive cells with Alexa488 azide was performed according to the manufacturer's protocol.
Instrument	BD FACS CANTOII flow cytometer (BD Biosciences)
Software	BD FACSDiva Software and FlowJo (FlowJo LLC)
Cell population abundance	Only flow cytometric analysis but no sorting was performed.
Gating strategy	From the initial cell population, which was gated in FSC-A/SSC-A by eliminating debris, singlets were discriminated using forward scatter (FSC-H/FSC-W) and side scatter (SSC-H/SSC-W) plots. Boundaries between positive and negative populations were defined using unstained or isotype controls. Representative gating strategies for each analysis are depicted in Supplementary Figures 5a and 6.

☒ Tick this box to confirm that a figure exemplifying the gating strategy is provided in the Supplementary Information.
